# JAM: A Scalable Bayesian Framework for Joint Analysis of Marginal SNP Effects

**DOI:** 10.1002/gepi.21953

**Published:** 2016-03-29

**Authors:** Paul J. Newcombe, David V. Conti, Sylvia Richardson

**Affiliations:** ^1^ MRC Biostatistics UnitCambridgeUnited Kingdom; ^2^ Division of BiostatisticsDepartment of Preventive MedicineZilkha Neurogenetic InstituteUniversity of Southern CaliforniaLos AngelesCaliforniaUnited States of America

**Keywords:** GWAS meta‐analysis, variable selection, fine‐mapping, glucose, insulin

## Abstract

Recently, large scale genome‐wide association study (GWAS) meta‐analyses have boosted the number of known signals for some traits into the tens and hundreds. Typically, however, variants are only analysed one‐at‐a‐time. This complicates the ability of fine‐mapping to identify a small set of SNPs for further functional follow‐up. We describe a new and scalable algorithm, joint analysis of marginal summary statistics (JAM), for the re‐analysis of published marginal summary stactistics under joint multi‐SNP models. The correlation is accounted for according to estimates from a reference dataset, and models and SNPs that best explain the complete joint pattern of marginal effects are highlighted via an integrated Bayesian penalized regression framework. We provide both enumerated and Reversible Jump MCMC implementations of JAM and present some comparisons of performance. In a series of realistic simulation studies, JAM demonstrated identical performance to various alternatives designed for single region settings. In multi‐region settings, where the only multivariate alternative involves stepwise selection, JAM offered greater power and specificity. We also present an application to real published results from MAGIC (meta‐analysis of glucose and insulin related traits consortium) – a GWAS meta‐analysis of more than 15,000 people. We re‐analysed several genomic regions that produced multiple significant signals with glucose levels 2 hr after oral stimulation. Through joint multivariate modelling, JAM was able to formally rule out many SNPs, and for one gene, *ADCY5*, suggests that an additional SNP, which transpired to be more biologically plausible, should be followed up with equal priority to the reported index.

## Introduction

Genome‐wide association studies (GWASs) have proved a hugely important tool in identifying novel regions of the genome underlying human disease and disease traits [Hindorff et al., 2009; Manolio, 2010; Visscher et al., 2012]. Recent large‐scale GWAS meta‐analyses have increased the number of unambiguous associations for some traits into the tens and even hundreds of regions [Lango Allen et al., 2010; Morris et al., 2012; Teslovich et al., 2010]. To power these discoveries, consortiums typically pull together numerous studies to amass a large number of individuals. For example, MAGIC (meta‐analysis of glucose and insulin related traits consortium) investigators identified three new loci associated with glucose levels 2 hr after an oral glucose challenge from a meta‐analysis with nine genome‐wide association studies and a total of over 15,000 individuals [Saxena et al., 2010]. More recently, more than 120,000 individuals from over 50 studies were used to identify 15 new breast cancer susceptibility loci [Michailidou et al., 2015]. The limited coverage of genome‐wide genotyping arrays makes it highly unlikely that the variants identified via GWAS are the true underlying causal variant for a given disease or trait. Current best practices now rely on imputing millions of common Single Nucleotide Polymorphisms (SNPs) across the genome to aid discovery. Once a region is identified, imputed data or sequenced data may be used to perform fine‐mapping studies to more thoroughly evaluate a region and attempt to identify the putative causal SNP or set of SNPs.

The availability of numerous, highly correlated variants presents several analytical challenges. In a typical GWAS, SNPs are only analysed one at a time. Often for large‐scale consortium meta‐analyses, this is all that is practically possible – each study performs a single SNP analysis utilising individual‐level data and summary association data are then shared with the consortium. This is a far from optimal statistical treatment of these valuable and expensive datasets. The main drawback, particularly relevant for subsequent fine‐mapping studies, is that single SNP analyses offer little insight into the number and location of causal variants at a given locus. In the presence of even moderate linkage disequilibrium (LD), many SNPs may produce significant associations. Multivariate joint models, which allow SNPs to be tested adjusted for other SNPs, can be used to identify which SNP or set of SNPs best represent the underlying signal. Indeed, there are several examples where joint analyses have led to the identification of secondary associations at a locus [Lango Allen et al., 2010; Ripke et al., 2011; Servin and Stephens, 2007; Sklar et al., 2011].

Ideally, we would re‐analyse published marginal GWAS results under a joint model. Unfortunately, when summary data only are available, the standard multivariate regression frameworks cannot readily be applied, because complete individual‐level data are required to evaluate the likelihood. For continuous and binary outcomes, respectively, Verzilli et al. [2008] and Newcombe et al. [2009] provide a Bayesian framework for formal multivariate analysis of summary data, including variable selection, using adapted likelihoods within which the unobserved association structure is imputed from a reference panel. However, these are relatively complex algorithms, implemented using full Markov Chain Monte Carlo (MCMC) sampling schemes of all parameters, and are only designed for the analysis of a modest number of SNPs. Hormozdiari et al. [2014], and building upon their work Chen et al. [2015], recently proposed approximate frameworks called CAVIAR and CAVIARBF, respectively, for multivariate re‐analysis of summary associations. Both frameworks use a Bayesian conjugate normal likelihood formulation with a plug‐in estimate for the residual error. The resulting analytical posterior allows efficient evaluation of the posterior support for all possible models up to a particular size. Benner et al. [2015] proposed a further extension named FINEMAP, which offers a stochastic search for when the number of possible models is prohibitively large. FINEMAP demonstrated equal but faster performance when the number of causal SNPs falls within the range evaluated by CAVIARBF, and improved performance when the true number of causal SNPs is higher. All these algorithms demonstrate the value of a sparse regression approach with summary data, however, they are designed for fine‐mapping applications and are not currently implemented for application to multiple regions at once. Another approximate approach, Probabilistic Identification of Causal SNPs (PICS), was recently proposed by Farh et al. [2014]. The algorithm takes the *P*‐value of the most highly associated (index) SNP in a region, and approximates adjusted statistics for other SNPs according to a simple linear function of their pairwise correlation in the 1,000 genomes reference data. Although asymptotically the trend between strength of association and LD might hold, in practice and in finite samples performance is unlikely to match formal multivariate modeling.

Based on the same likelihood first described by Verzilli et al. [2008], Yang et al. [2012] describe a frequentist inferential procedure for inferring adjusted SNP associations from marginal test statistics. Their procedure is designed to scale to a large number of SNPs, however, variable selection is performed through a simple stepwise procedure. Even when full individual‐level data are available, performing variable selection across large numbers of correlated predictors remains a very challenging problem. Traditional stepwise selection procedures are well known to be both conservative and unstable, often getting stuck at local maxima in the model space and leading to potentially spurious selections [Hocking, 1976; Miller, 2002; Tibshirani, 1996]. Consequently, Yang et al.'s [2012] framework restricts the estimation of multivariate models to SNPs with a maximum correlation of 0.9, leaving open the question of developing a scalable method based on summary statistics that can deal with the stronger LD structures necessary for fine‐mapping. Ideally we might use more sophisticated approaches including penalised regression, an area in active development under a frequentist [Tibshirani, 1996; Tibshirani et al., 2005; Zou, 2006; Zou and Hastie, 2005], and Bayesian framework [Bottolo et al., 2011, 2013; Bottolo and Richardson, 2010] for performing variable selection among numerous and highly correlated predictors. These approaches have proven successful in a number of genomic examples [Bottolo et al., 2011, 2013; Peng et al., 2010; Vignal et al., 2011; Wu et al., 2009].

In this work, we present JAM (joint analysis of marginal associations), a novel and scalable algorithm for multivariate analysis of summary associations. Models and SNPs that best explain the joint pattern of marginal effects over all SNPs in a region are highlighted via a Bayesian regression framework that integrates priors on the SNP effects and model space. This offers robust and efficient variable selection allowing application in the presence of strong LD to support fine‐mapping work. SNP effects are conditioned on one another by accounting for correlation as estimated from a reference panel, in the spirit of Verzilli et al. [2008], Yang et al. [2012], CAVIARBF, FINEMAP, and other approaches. We have derived a novel conjugate form of the multivariate likelihood by invoking a Cholesky transformation to a set of independent Gaussian distributions, leading to an extremely efficient algorithm in which all parameters are integrated out, including the residual. This contrasts with other frameworks such as CAVIARBF and FINEMAP in which the residual is fixed. In combination with a haplotype block decomposition, computational time increases only linearly with the number of covariates. JAM proceeds by enumerating all models including up to three causal SNPs per region, but we have also implemented a more general version using Reversible Jump MCMC for when the number of causal SNPs may be larger or is unknown.

In a series of realistic simulation studies with real‐life strong patterns of LD, we start by demonstrating equivalent performance of JAM to related approaches CAVIARBF and FINEMAP in a single region setting. We then explore performance of JAM applied to multiple regions involving up to 10,000 SNPs. JAM consistently demonstrated increases in the number of true signals among top ranked associations compared to Yang et al.'s [2012] COJO stepwise selection procedure which, to our knowledge, is the only other multivariate software designed for application to summary statistics from multiple regions. Analysis of 10,000 SNPs in JAM took less than 3 hr using Reversible Jump MCMC. We also present a real data application of the published marginal results from MAGIC, in which we re‐analysed several genomic regions associated with 2 h fasting glucose. Inference from our multivariate JAM framework considerably reduces the number of SNPs to consider following up, and for one gene, *ADCY5*, joint modelling of the complete pattern of marginal effects suggests another SNP should be followed up with equal priority to the reported GWAS index.

## Methods

### Multivariate Normal Model for Summary Data

We start by deriving the likelihood of the reported marginal SNP associations as a function of the unobserved effects conditioned on one another, as described by Verzilli et al. [2008]. Define y as an *N*‐length vector of phenotype values. Under the standard linear regression model when full individual data is available, the relationship between y and the *P* SNPs may be modelled as:

(1) $$y∼N(X\beta ,\sigma^{2}I_{N}),$$

where X is the N×P design matrix containing each individual's genotype. Note that as in Yang et al. [2012], for efficiency, we mean centre the outcome, y, and covariates, so as to avoid fitting an intercept term. Under, for example, an additive model, elements of X corresponding to marker *m* are thus mean‐centred to $-2p_{m}$, $1-2p_{m}$, or $2-2p_{m}$, where $p_{m}$ is the minor allele frequency (MAF). $\beta =(\beta_{1},...,\beta_{P})$ is the *P*‐length vector of multivariate SNP effects, that is, adjusted for one another. $I_{N}$ is the N×N identity matrix and σ^2^ is the residual variance (assumed the same for every individual).

GWASs, typically only report and/or share summary associations from univariate one‐at‐a‐time tests. Suppose that for each marker *m*, we only have access to its univariate effect estimate, ${\hat{\beta }}_{m}$ and MAF, ${\hat{p}}_{m}$. If we define a *P*‐length vector z as $z=X^{{}^{\prime }}y$, then all *P* entries of z can be estimated from these summary association data. First, assuming Hardy Weinberg Equilibrium and an additive genetic model, we construct estimates of the genotype counts $n_{mg}$ for g=0,1,2, and trait mean within each genotype group ${\bar{y}}_{mg}$ for each marker *m*, as described in the supplementary Methods. The *m*th entry of z, corresponding to SNP *m*, is then given by:

(2) $$z_{m}=\sum_{i}y_{i}\times x_{m,i}^{{}^{\prime }}={\bar{y}}_{m1}n_{m1}+2{\bar{y}}_{m2}n_{m2},$$

where $x_{m,i}^{{}^{\prime }}$ is the design matrix entry corresponding to individual *i*'s genotype for SNP *m*. Note that this approximate method of obtaining z from effect estimates was used in the simulation and case studies below, and does not appear to have a meaningful impact on performance. Therefore, the *m*th element of z may be thought of as the total trait burden across all risk alleles of SNP *m*, which are present in the study population. Using Equation (1) we can derive the distribution of z using standard linear algebra (according to the transformation $X^{{}^{\prime }}$):

(3) $$z∼MVN_{P}\left(X^{{}^{\prime }}X\beta ,\sigma^{2}X^{{}^{\prime }}X\right)$$

Thus, the multivariate normal likelihood of the summary data, z, can be expressed in terms of the multivariate adjusted SNP effects ***β***. Because the genotype matrix X is unobserved, we propose constructing a plug‐in estimate for $X^{\prime }X$ in (3) from a reference panel, such as the Welcome Trust Case Control Consortium (WTCCC) [2007] or the 1,000 genomes project [The 1000 Genomes Project Consortium, 2010], in the spirit of Verzilli et al. [2008] and Yang et al. [2012] – see the Supplementary Methods for details.

#### Cholesky Decomposition Transformation

JAM incorporates several novel developments for computing the likelihood described above. The first of these is the use of a Cholesky decomposition to transform the data to a vector of independent statistics. Because $X^{\prime }X$ is inherently Hermitian (equal to its own transpose), if it is also positive definite then we can perform a Cholesky decomposition:

$$X^{\prime }X=L^{\prime }L,$$

where L is an upper triangular P×P matrix with positive diagonal elements, and hence invertible. Transforming z by the Cholesky transpose inverse $L^{\prime -1}$ we obtain a distribution of independent observations:

(4) $$\begin{array}{ccc} L^{\prime -1}z & ∼ & MVN_{P}\left(L^{\prime -1}X^{\prime }X\beta ,\sigma^{2}L^{\prime -1}X^{\prime }XL^{-1}\right)\\ & ∼ & MVN_{P}\left(L^{\prime -1}L^{\prime }L\beta ,\sigma^{2}L^{\prime -1}L^{\prime }LL^{-1}\right)\\ & ∼ & MVN_{P}\left(L\beta ,\sigma^{2}I_{P}\right). \end{array}$$

Working with a vector of independent Gaussian statistics substantially simplifies the computational requirements for JAM. Henceforth, for convenience, we denote the Cholesky decomposition transformed (independent) marginal statistics as $z_{L}=L^{\prime -1}z$.

### Bayesian Model Selection Formulation

Yang et al. [2012] use the multivariate normal likelihood defined in (3) to infer adjusted SNP *p*‐values according to the corresponding maximum likelihood estimates and standard errors. Model selection then proceeds by adding/removing SNPs and re‐estimating *p*‐values according to a stepwise procedure. To avoid stepwise selection issues, JAM invokes a Bayesian variable selection procedure. Rather than fixing which SNPs to include, we wish to treat the model, which we denote using a *P*‐length vector of indicators for each SNP, γ, as unknown. Denote $\beta_{\gamma }$ as the sub‐vector of non‐zero effects extracted from ***β***. Conditional on model γ the likelihood (4) becomes:

(5) $$p\left(z_{L}|\gamma ,\beta_{\gamma },\sigma^{2}\right)=MVN_{P}\left(L_{\gamma }\beta_{\gamma },\sigma^{2}I_{P}\right),$$

where $L_{\gamma }^{\prime }$ is the sub‐matrix of $L^{\prime }$, containing only the columns corresponding to co‐variates included in γ. Note in (5) that regardless of which SNPs are selected in γ, the complete vector of summary statistics, $z_{L}$, is always modelled. This represents an important difference with Yang et al.'s [2012] framework in which data are only modelled for SNPs included in the current model; we borrow information across the complete set of marginal effects at all times.

The second major development incorporated in JAM is to formally define the conjugate posterior for the marginal statistics. Because we have transformed the summary data to a vector of independent statistics, observing that (5) is of the same form as a traditional linear model, the conjugate normal structure of ($\beta_{\gamma },\sigma^{2}$) therefore follows a normal‐inverse‐gamma distribution:

(6) $$p\left(\beta_{\gamma }|\sigma^{2},\gamma \right)=MVN_{P}\left(m_{\gamma },\sigma^{2}\Sigma_{\gamma }\right)$$

(7) $$p\left(\sigma^{2}|\gamma \right)=P\left(\sigma^{2}\right)=InvGa\left(a_{\sigma },b_{\sigma }\right).$$

In all analyses below, we chose $a_{\sigma }=b_{\sigma }=0.01$, a relatively uninformative setup. With some additional conditional independence assumptions, the joint posterior may be expressed as:

$$p\left(\beta_{\gamma },\sigma^{2},\gamma |z_{L}\right)\propto p\left(z_{L}|\beta_{\gamma },\sigma^{2},\gamma \right)p\left(\beta_{\gamma }|\sigma^{2},\gamma \right)p\left(\sigma^{2}|\gamma \right)p\left(\gamma \right).$$

Exploiting the conjugate prior setup (6) and (7), Brown et al. [1998] show how ***β*** and σ may be integrated out leading to a closed form expression for the marginal posterior of γ:

(8) $$\begin{array}{ccc} p(\gamma |z_{L}) & \propto & \int p\left(\beta_{\gamma },\sigma^{2},\gamma |z_{L}\right)d\beta_{\gamma }d\sigma^{2}\\ & = & \int p\left(z_{L}|\beta_{\gamma },\sigma^{2},\gamma \right)p\left(\beta_{\gamma }|\sigma^{2},\gamma \right)p\left(\sigma^{2}|\gamma \right)d\beta_{\gamma }d\sigma^{2}\\ & \propto & |L_{\gamma }^{\prime }L_{\gamma }+\Sigma_{\gamma }^{-1}{|}^{-1/2}{\left|\Sigma_{\gamma }\right|}^{-1/2}{\left(2b_{\sigma }+S\left(\gamma \right)\right)}^{-(2a_{\sigma }+P-1)/2}, \end{array}$$

where $S\left(\gamma \right)=C-M^{\prime }K_{\gamma }^{-1}M$ with $C={\left(z_{L}\right)}^{\prime }\left(z_{L}\right)+m_{\gamma }^{\prime }\Sigma_{\gamma }^{-1}m_{\gamma }M=L_{\gamma }^{\prime }\left(z_{L}\right)+\Sigma_{\gamma }^{-1}m_{\gamma }$, and $K_{\gamma }=L_{\gamma }^{\prime }L_{\gamma }+\Sigma_{\gamma }^{-1}$ [Bottolo and Richardson, 2010; Brown et al., 1998].

We adopt a so‐called *g*‐prior formulation for the SNP effects $\beta_{\gamma }$, and set $m_{\gamma }=0$ and $\Sigma_{\gamma }=\tau {\left(L_{\gamma }^{\prime }L_{\gamma }\right)}^{-1}$ in (6). Because ${\left(L^{\prime }L\right)}^{-1}={\left(X^{\prime }X\right)}^{-1}$, SNP effects are therefore ascribed correlation structure and ranges of supported effects inversely proportional to their corresponding co‐variances and variances, respectively, in the observed genotype matrix X. τ is an unknown parameter that controls the magnitude of this proportional relationship (and consequently the shrinkage). There is a substantial literature discussing the benefits of a *g*‐prior formulation and choices for τ [Cui and George, 2008; Fernández et al., 2001; Liang et al., 2008]. Because our main goal is algorithmic efficiency, we choose to treat τ as a constant and follow suggestions to select a value equal to the maximum of *P* ^2^ and *N* [Fernández et al., 2001; George and McCulloch, 1997]. An attractive property of the *g*‐prior is the resulting simplified marginal likelihood (8) which becomes:

(9) $$\begin{array}{ccc} & & \;\;p\left(z_{L}|\gamma \right)\propto |L_{\gamma }^{\prime }L_{\gamma }+L_{\gamma }^{\prime }L_{\gamma }{/\tau |}^{-1/2}{\left|\tau {\left(L_{\gamma }^{\prime }L_{\gamma }\right)}^{-1}\right|}^{-1/2}\\ & & \;\;{\left(2b_{\sigma }+S\left(\gamma \right)\right)}^{-(2a_{\sigma }+P-1)/2}={\left|\left(\tau +1\right)I_{p_{\gamma }}\right|}^{-1/2}\\ & & \;\;{\left(2b_{\sigma }+S\left(\gamma \right)\right)}^{-(2a_{\sigma }+P-1)/2}={\left(\tau +1\right)}^{-p_{\gamma }/2}{\left(2b_{\sigma }+S\left(\gamma \right)\right)}^{-(2a_{\sigma }+P-1)/2}, \end{array}$$

where

$$S\left(\gamma \right)={\left(z_{L}\right)}^{\prime }\left(z_{L}\right)-\frac{\tau }{1+\tau }{\left(z_{L}\right)}^{\prime }L_{\gamma }{\left(L_{\gamma }^{\prime }L_{\gamma }\right)}^{-1}L_{\gamma }^{\prime }z_{L}.$$

Finally, the JAM model is completed by specifying the prior distribution over models, p(γ). Following Scott and Berger [2010], we adopt a beta‐binomial setup whereby an unknown hyper‐parameter, ω, is introduced as the proportion of ‘true’ effects. This is assigned a beta hyper‐prior:

$$\omega ∼Beta(a_{\omega },b_{\omega }).$$

Conditional on ω, a binomial prior is assumed over the model size, assuming all models of the same dimension are equally likely. Therefore:

(10) $$p\left(\gamma \right)\;=\;\int P\left(\gamma |\omega \right)P\left(\omega \right)\;=\;\frac{B(\gamma^{{}^{\prime }}I_{P}+a_{\omega },P-\gamma^{{}^{\prime }}I_{P}+b_{\omega })}{B(a_{\omega },b_{\omega })},$$

where *B* is the Beta function. For most analyses below, we used a beta‐binomial prior of $a_{\omega }=1$, $b_{\omega }=9$, which corresponds to a weakly informative prior on the proportion of truly casual SNPs centred on 10%, and resulted in performance as expected under the null (see supplementary Fig S1). Combining (9) and (10), we can express the marginal posterior of models, γ, as:

(11) $$p\left(\gamma |z_{L}\right)\propto p\left(\gamma \right)p\left(z_{L}|\gamma \right).$$

#### Block Independence Decomposition

For invertibility of the $X^{\prime }X$ plug‐in estimate, we require a full rank X genotype matrix from the reference panel. When working with a large number of SNPs, it is convenient to split the covariates into *B* blocks, $X_{b}$, between which we can assume independence – e.g. if the blocks correspond to LD blocks – and within each of which ${X_{b}}^{\prime }X_{b}$ is invertible. Decomposing the likelihood (9) into a product, the posterior for γ in Equation (11) becomes:

$$p\left(\gamma |z_{L}\right)\propto p\left(\gamma \right)\prod_{b=1}^{B}p\left(L_{b}^{-1}z_{b}|\gamma_{b}\right),$$

where $z_{b}$ and $\gamma_{b}$ are the summary statistic and model sub‐vectors, respectively, corresponding to SNPs in block *b*, and $L_{b}$ is derived from the Cholesky decomposition corresponding to $X_{b}$.

We suggest partitioning large genomic regions of interest into approximately independent blocks via the haplotype block recognition algorithm first proposed by Gabriel et al. [2002]. Implementations exist in the widely used software Haploview [Barrett et al., 2005], and Plink [Purcell et al., 2007]. Researchers may also wish to consider the recent and substantially more efficient MIG++ implementation [Taliun et al., 2014]. Some experimentation will be required with the Haplotype block recognition parameters to ensure that each block of the resulting partition corresponds to a full rank genotype matrix in the reference data. Inherently no block can be larger than the reference sample size. In our fine‐mapping case study, after LD pruning for a maximum correlation of 95%, all LD blocks of interest were less than 100 SNPs long, and full rank genotype matrices were readily available from our reference sample of size 2,674. As larger reference samples such as the UK10K are fast becoming available, our method will be applicable to denser correlation structures and larger blocks. This will, however, inherently require the inversion of increasingly large X'X matrices. Recently the spectral decomposition has been leveraged in high dimensional genomics linear mixed models to reduce the computational cost of inverting large correlation matrices from cubic to linear complexity [Canela‐xandri et al., 2015; Kang et al., 2008; Speed and Balding, 2014]. In the future, we plan to implement the same decomposition into JAM, readying the algorithm for scalability to larger LD blocks.

### Posterior Model Inference

To measure the importance of a particular model γ∈Γ, we seek to estimate the posterior probability:

(12) $$p\left(\gamma |z_{L}\right)=C^{-1}p\left(\gamma \right)p\left(z_{L}|\gamma \right),$$

where *C* is the normalising constant, equal to the posterior mass over all possible models:

$$C=\sum_{\gamma \in \Gamma }p\left(\gamma \right)p\left(\gamma |z_{L}\right).$$

For a particular model, γ, using the expressions provided in (9) and (10), the posterior and prior support under JAM may be calculated. If we only consider models that include up to, say, three SNPs, then if the number of total SNPs *P* is modest, it is computationally fast to evaluate (9) and (10) for all 1+P + P2 + P3 possible models. For example, if P=100, this corresponds to 166,850 calculations, which our algorithm achieves in seconds. Thus, we obtain an estimate of *C*:

$$\hat{C}=\sum_{\gamma \in \Gamma^{*}}p\left(\gamma \right)p\left(\gamma |z_{L}\right),$$

where $\Gamma^{*}\subseteq \Gamma$ denotes the truncated model space obtained by excluding models of dimension higher than 3. Approximate posterior probabilities (APPs) for all models in $\Gamma^{*}$ may then be obtained substituting $\hat{C}$ into (12). This procedure can be efficiently extended to large numbers of SNPs if the block independence decomposition described in Section Bayesian Model Selection Formulation is invoked, and independent but calibrated beta‐binomial priors are placed on the dimension within each block (see the supplementary Methods for details). Analysis of 10,000 SNPs decomposed into the realistic LD blocks described in the simulation study took less than 3 min (see supplementary Fig. S2).

If models including four or more SNPs within each gene/block may be assumed extremely unlikely, i.e. the vast majority of posterior mass over Γ is contained in $\Gamma^{*}$, this approximate inferential approach should provide reasonable posterior inference under JAM without the need for Markov Chain Monte Carlo sampling. This assumption seems reasonable, and posterior inference via enumeration in similarly truncated spaces has proven effective under various frameworks designed for genetic association analysis [Hormozdiari et al., 2014; Kichaev et al., 2014; Servin and Stephens, 2007].

Our software allows the maximum model size to be increased, which would be advisable for sensitivity analyses in a limited number of regions of particular interest, or when prior biology points to the existence of more signals. As a generic extension, we also provide details for and have implemented a simple add/delete/swap Reversible Jump algorithm for MCMC sampling of models (see the supplementary Methods), which can deal with greater model uncertainty and does not restrict the model space to a small fixed number of causal SNPs in advance. When using the stochastic Reversible Jump model search, convergence should be checked and assessed using the built in variable selection autocorrelation plots (see, e.g. supplementary Fig. S7), increasing the number of iterations as necessary, and comparing results across different MCMC seeds.

### Posterior Inference of Adjusted SNP effects

Having selected a model, or if researchers are interested in a particular SNP or set of SNPs a priori, we provide a method for making posterior inference on the corresponding multivariate adjusted effects under JAM. Conditioning on a model γ, i.e. the selection of SNPs of interest, due to the conjugate Normal‐inverse‐Gamma structure of our framework, it is possible to define the joint normal‐inverse‐Gamma posterior distribution of $\beta_{\gamma }$ and σ^2^ as:

(13) $$\begin{array}{c} p\left(\sigma^{2}|z_{L},\gamma \right)=InvGa\left(a_{\sigma }+\frac{P}{2},b_{\sigma }+\frac{s^{2}}{2}+\frac{{\hat{\beta }}_{\gamma }L_{\gamma }^{\prime }L_{\gamma }{\hat{\beta }}_{\gamma }}{2(\tau +1)}\right), \end{array}$$

(14) $$p\left(\beta_{\gamma }|z_{L},\sigma^{2},\gamma \right)=MVN\left(\frac{\tau {\hat{\beta }}_{\gamma }}{1+\tau },\frac{\tau \sigma^{2}{\left(L_{\gamma }^{\prime }L_{\gamma }\right)}^{-1}}{1+\tau }\right),$$

where ${\hat{\beta }}_{\gamma }={\left(L_{\gamma }^{\prime }L_{\gamma }\right)}^{-1}L_{\gamma }^{\prime }z_{L}$ and $s^{2}={\left(z_{L}-L_{\gamma }{\hat{\beta }}_{\gamma }\right)}^{\prime }\left(z_{L}-L_{\gamma }{\hat{\beta }}_{\gamma }\right)$. Posterior samples of $\left(\beta_{\gamma },\sigma^{2}\right)$ are hence straight forward to generate using a Gibbs sampler to first draw σ^2^ from the inverse‐Gamma distribution defined in (13), then draw $\beta_{\gamma }$ after plugging the σ^2^ draw into the multivariate Normal distribution defined in (14). This is implemented in our R package.

## Results

Our Results section is split into a simulation study followed by a case study in which JAM was used to re‐analyse results for several candidate genes associated with 2‐hr glucose levels after oral stimulation published by the MAGIC consortium. All simulations were designed to have the same number of individuals and genetic correlation structures observed in the MAGIC case study.

### Simulation Study

#### Comparison of JAM With Other Fine‐Mapping Strategies in a Single Region

We compare the performance of JAM to the fine‐mapping frameworks CAVIARBF and FINEMAP, which also facilitate multivariate sparse Bayesian model selection using summary statistics, as well as Yang et al.'s stepwise selection framework (COJO within the GCTA software package). Conceptually, CAVIARBF and FINEMAP are very similar to JAM, the main difference being that they fix the residual error at an assumed value rather than treat it as unknown. Using the WTCCC genotype matrix for the *TCF7L2* region (41 SNPs, n=2,674, Affymetrix 500K), we bootstrapped rows to obtain a genotype matrix for n=15,356 individuals. Multivariate modelling will offer benefits when the genetic architecture is more complex than a single SNP effect within a region. Therefore, we simulated outcome data under a multi‐SNP linear regression model with one main effect at the reported index (and of the same size), a secondary effect at a weakly correlated SNP, and a tertiary effect at an inverse correlated SNP. The residual error was set to 0.97, the standard deviation in 2‐hr glucose reported by MAGIC. The correlation structure over the region is shown in supplementary Figure S5.

CAVIARBF, FINEMAP and JAM's model enumeration procedure were all setup to consider causal configurations including up to three SNPs. FINEMAP was passed the marginal *z*‐scores and run with default values. CAVIARBF was passed the marginal effects and the actual residual variance used in the simulations, whereas JAM used a weakly informative inverse‐Gamma (0.01, 0.01) prior on the residual variance, as explained in the methods. COJO was set to use a Bonferroni significance threshold for the number of SNPs (p=0.0012). All methods were provided with the marginal one‐at‐a‐time SNP effects, and the same ‘reference dataset’ consisting of n=2,674 independently bootstrapped rows (i.e. the number of WTCCC controls). We do not compare against CAVIAR, because CAVIARBF has already been shown to be equally accurate but more efficient [Chen et al., 2015]. We have not included PICS in the simulation study because the authors do not share a scriptable algorithm. Use of PICS is restricted to manual submission of single summary statistics into a webpage, with no choice of reference dataset other than the integrated 1,000 genomes.

**Figure 1:**
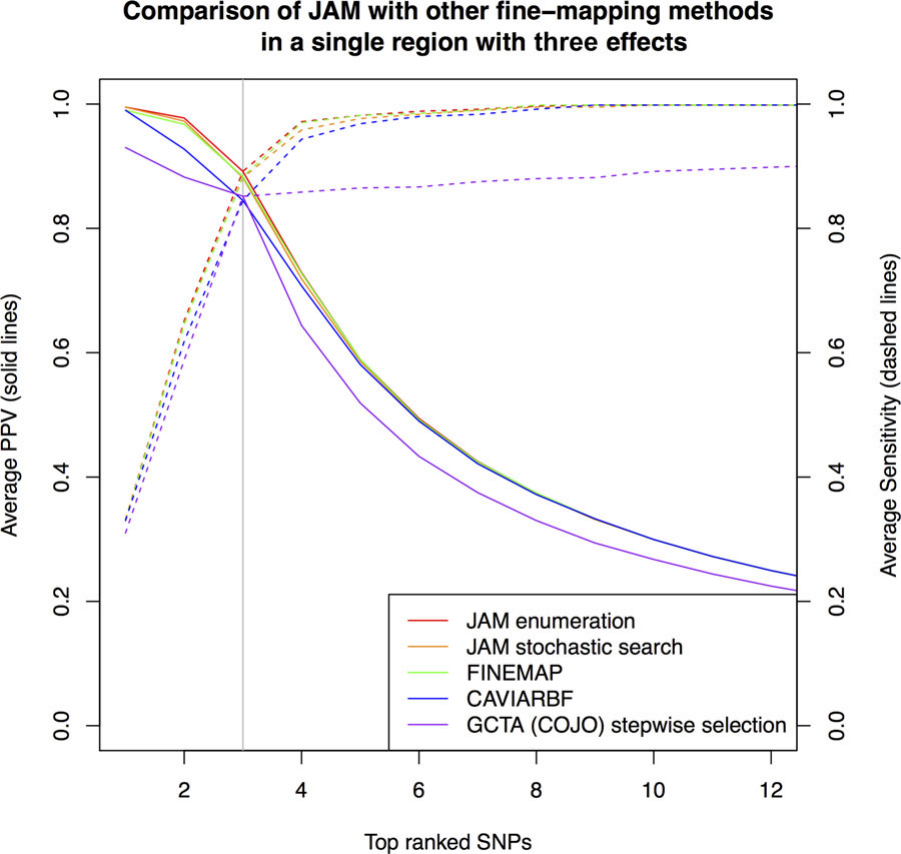
Comparison of ranking performance by JAM against other fine‐mapping strategies for 15,356 individuals (the total size of the MAGIC consortium). Ranking performance is measured in terms of PPV, the proportion of true signal SNPs in the selection (solid lines, left *y*‐axis) and power/sensitivity, the proportion of all simulated signals included (dashed lines, right *y*‐axis). For each method, the average PPV and sensitivity estimates consist of points for each SNP rank, which we have joined with lines to ease the visual comparison. Data were simulated for a single region of 41 SNPs, three of which were given effects as described in the main text. For LD estimation, JAM, FINEMAP, CAVIARBF and GCTA (COJO) were provided with an independently simulated reference dataset of 2,674, the size of the WTCCC control sample. Estimates are averaged over 200 simulation replicates. A vertical grey line highlights the rank equal to the number of true signals, where PPV and sensitivity by definition intersect. Performance of JAM's enumeration (red), JAM's stochastic search (orange) and FINEMAP (green) were indistinguishable and hence these lines are superimposed on top of one another. Performance of CAVIARBF (blue) was marginally weaker than JAM and FINEMAP for top‐ranked SNPs, but indistinguishable at lower ranks.

Figure 1 shows the proportion of true signals included among top‐ranked SNPs (positive predictive value (PPV), left *y*‐axis), as well as the power/sensitivity (right *y*‐axis), over a range of rank thresholds, and averaged over 200 simulation replicates. The sparse Bayesian regression approaches (JAM, CAVIARBF and FINEMAP) all performed very similarly, offering near perfect discrimination of the three signal SNPs. They all outperformed COJO's stepwise search. As a proof of principle and for the sake of comparison, we ran JAM's stochastic search, which does not require an assumption on the maximum number of causal SNPs and resulted in very similar performance to inference via exhaustive evaluation of models with three SNPs or less. The stochastic search was run for two million iterations, and convergence was assessed using different MCMC seeds and by inspecting variable selection trace plots for a random subset of simulations (e.g. see supplementary Fig. S7).

#### Comparison of JAM With Stepwise Selection Across Multiple Regions

Next, we explored performance of JAM in an analysis of multiple regions simultaneously. We considered two scenarios. The first is analogous to our case study below and considers the same four candidate regions. Data were simulated for n=15,356 individuals as above, but now according to a genotype matrix across all four regions from our case study (132 SNPs total). Multi‐SNP signals were simulated in each region, as described above, and ranged in size between half and double the largest effect reported by MAGIC (0.13). Supplementary Figure S5 displays heatmaps of the four regions, and where the simulated signals were placed. This simulation scenario, with four loci all of which have effects, corresponds to settings when a limited number of candidate regions would be the focus of a multivariate re‐analysis of GWAS results, such as in fine‐mapping of multiple GWAS regions. To demonstrate the scalability of our approach, we also explored a ‘needle in the haystack’ scenario by duplicating these regions many times up to 10,000 SNPs, but simulating outcomes under the same model, with effects only placed in four regions. Two hundred replicate datasets were simulated for each scenario.

We again compare results against Yang et al.'s [2012] multivariate COJO stepwise search. The summary statistic fine‐mapping frameworks (FINEMAP, CAVIARBF and CAVIAR) are not currently implemented for application to multiple regions simultaneously, so are not included in the comparisons below. Given the similarities with these frameworks, and performance in the single region setting, we henceforth consider JAM to represent this general class of approach ‐ sparse Bayesian multivariate regression of summary statistics. We also compared against univariate approximate Bayes Factors, proposed by Wakefield [2009], and their corresponding Approximate Posterior Probabilities (APPs) described by Maller et al. [2012]. These improve upon the naive ranking of marginal *p*‐values by incorporating prior information on the range of effect sizes, and that each individual SNP is very unlikely to have an effect. Note that comparatively to JAM and COJO, the APPs do not provide multivariate inference; they are not LD adjusted and are inferred under an assumption of no more than one effect per region. We calculated APPs according to a *N*(0, 0.2) prior on each SNP, which corresponds to 95% weight on a range of values up to 50% larger than the maximum simulated effect magnitude, and with a sparsity inducing prior probability of $10\times 10^{-4}$ on each SNP, as is typically used. For the purpose of benchmarking, we also present results from an ‘optimal’ treatment of each simulated dataset, in which it was analysed under an analogous Bayesian linear model selection framework for complete individual patient data (IPD), using identical priors to those in JAM. Because merging all consortium data for full multivariate analysis of IPD is usually unrealistic due to logistics and/or data sharing arrangements, we also present results from a subset analysis – analogous to one of the contributing cohorts choosing to perform multivariate adjusted analysis of their own data in isolation. Sub‐cohorts of size n=1,706, the average MAGIC sub‐cohort size, were randomly sampled each replicate. For JAM and the Bayesian IPD penalised regression analyses, regions were analysed under the block independence assumption detailed in the methods.

**Figure 2:**
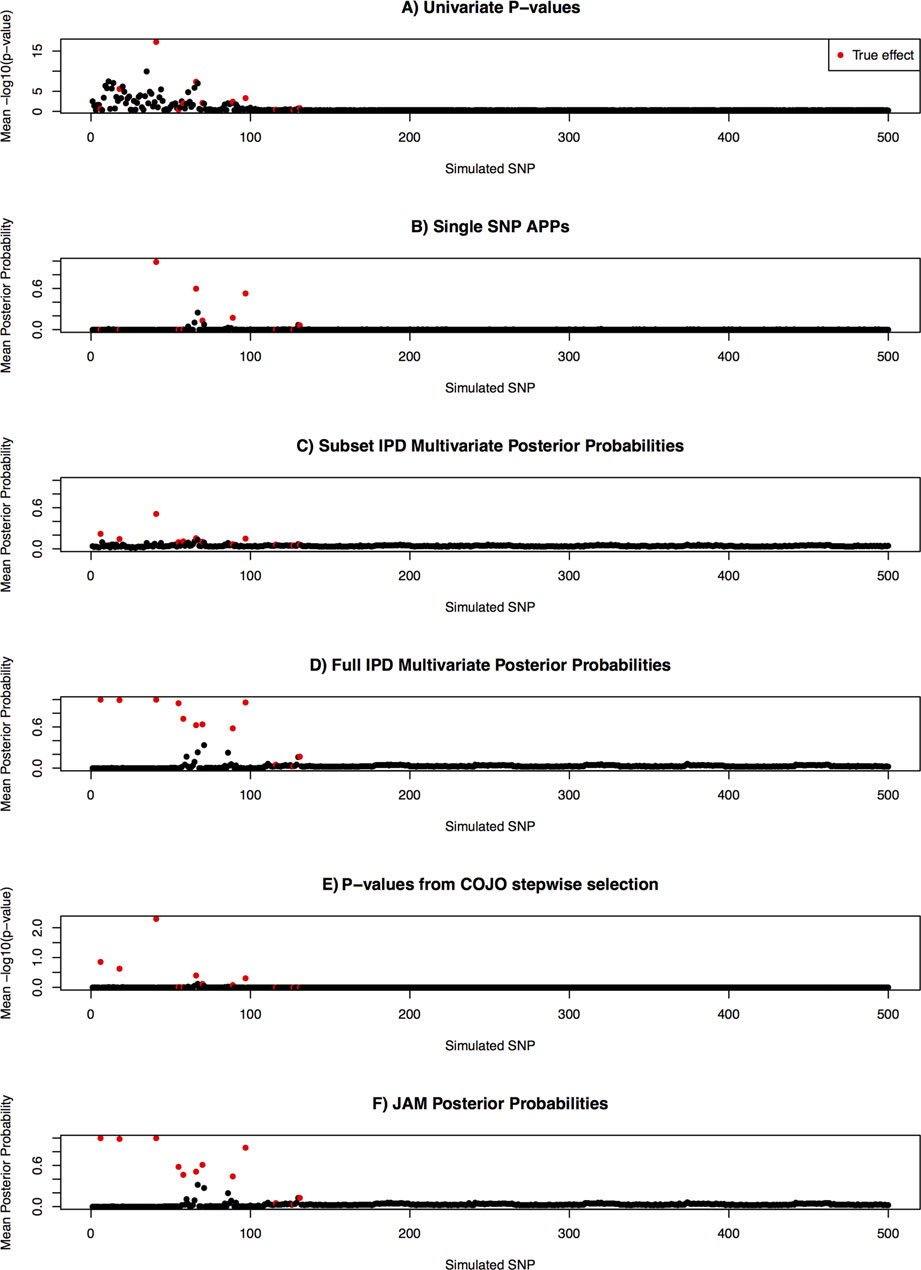
Comparison of signal to noise discrimination by JAM against various strategies when 12 effects were simulated among 10,000 SNPs for 15,356 individuals (the total size of the MAGIC consortium). Results are only displayed for the first 500 simulated SNPs, which included all 40 simulated effects. For LD estimation, JAM was provided with an independently simulated reference dataset of 2,674, the size of the WTCCC control sample. All summary statistics are averaged over 200 simulation replicates. The 12 true effects are highlighted in red. IPD: individual patient data; ABF: Wakefield's approximate Bayes factor.

Figure 2 shows average SNP statistics from the various strategies under the 10,000 SNP simulation scenario. The univariate APPs improved upon the marginal *P* values to clean up much of the noise due to LD among the top associations, owing to the incorporation of sparsity inducing priors. However, due to the lack of multivariate adjustment and the assumption of a single effect per region, secondary effects were usually underestimated, or missed completely. Through formal multivariate modelling, JAM and COJO were able to identify additional effects within the causal regions. JAM, however, demonstrated considerably better discrimination, resulting in performance close to the ‘optimal’ complete IPD multivariate analysis if the original data, rather than summaries, were available. The subset IPD multivariate analysis clearly suffers from a lack of power.

**Figure 3:**
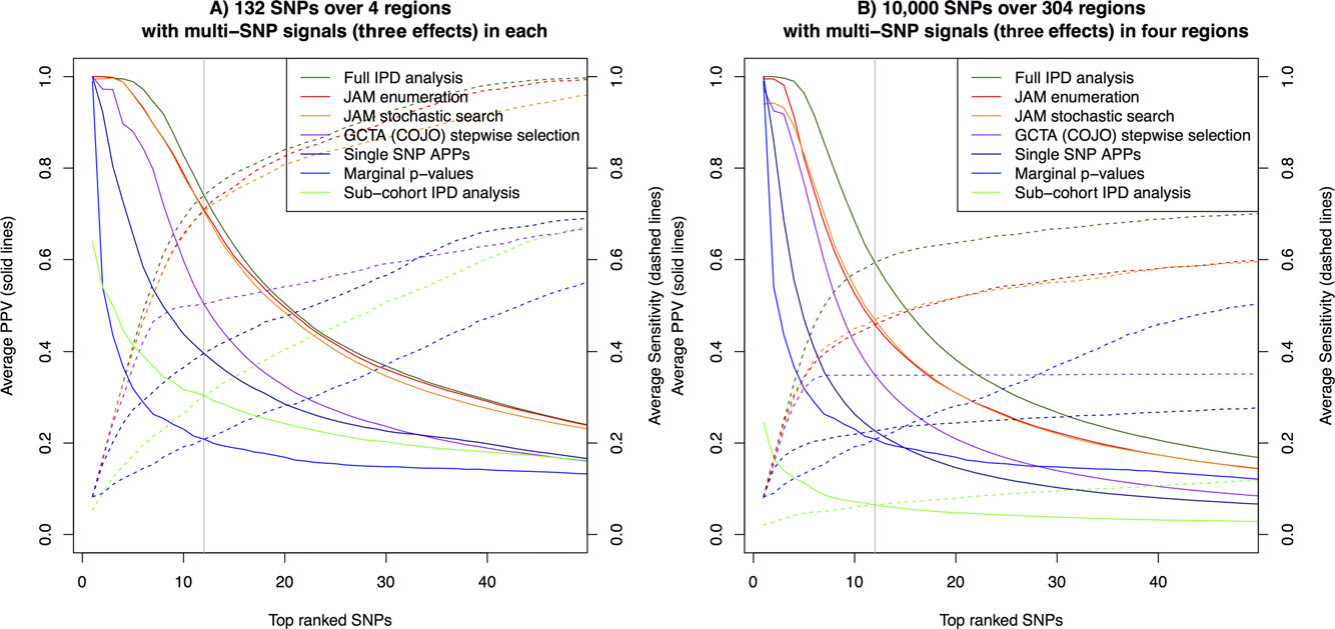
Comparison of ranking performance by JAM against various other strategies across two simulation scenarios for 15,356 individuals (the total size of the MAGIC consortium). Ranking performance is measured in terms of PPV, the proportion of true signal SNPs in the selection (solid lines, left *y*‐axis) and power/sensitivity, the proportion of all simulated signals included (dashed lines, right *y*‐axis). For each method, the average PPV and sensitivity estimates consist of points for each SNP rank, which we have joined with lines to ease the visual comparison. Panel (A) corresponds to a fine‐mapping scenario, including 132 SNPs across four regions, and panel (B) corresponds to a higher dimensional setting in which 40 effects were simulated among 10,000 SNPs. For LD estimation, JAM and GCTA (COJO) were provided with an independently simulated reference dataset of 2,674, the size of the WTCCC control sample. Estimates are averaged over 200 simulation replicates. A vertical grey line highlights the rank equal to the number of true signals, where PPV and sensitivity by definition intersect. IPD: individual patient data; ABF: Wakefield's approximate Bayes factor. Performance of JAM's enumeration (red), JAM's stochastic search (orange) were nearly identical in both scenarios, and so these lines appear superimposed. In the four region scenario, JAM's performance was very similar to the full IPD data analysis (dark green), and caught up at lower ranks at which point this line also appears superimposed.

Figure 3 shows the proportion of true signals included among top‐ranked SNPs (PPV, left *y*‐axis), as well as the power/sensitivity (right *y*‐axis), over a range of rank thresholds for the multi‐region simulation scenarios. In both the four region scenario, and the harder 10,000 SNP scenario, JAM offered gains in PPV and sensitivity over the marginal *p*‐values, single SNP APPs and the COJO multivariate stepwise selections. Therefore, top SNPs identified by JAM were more likely to contain either true signal SNPs (or in practice the best tag among the SNPs genotyped). The sub‐cohort multivariate IPD analyses, which analyse just 1/9 of the data, performed poorly again, and generally worse than the single SNP statistics.

Despite the much larger model space, JAM's stochastic search continued to offer similar performance to inference via enumeration of configurations in each block up to dimension three. The stochastic search took longer to run (41 vs. 4 sec for two million iterations in the four region scenario; 3 hr vs. 5 min for 20 million iterations in the 10,000 SNP scenario), but it avoids making an assumption on the maximum number of causal SNPs. Therefore, despite the longer run times, we recommend the stochastic search as the default option for inference. Convergence was again assessed using different random seeds and through inspection of variable selection trace plots (e.g. see supplementary Fig. S8).

#### Detection of Multi‐SNP Models

In addition to improving discrimination of signal to noise, it is of interest whether JAM can extract the underlying number of effects within a region from summary data. Focusing on the simulated gene with average effect magnitudes, we compared Bayes factors for ⩾1, 2 and 3 signals between data simulated under single SNP and multi‐SNP models. When one effect was simulated, JAM picked it up decisively and, encouragingly, suggested no evidence for more than one effect. When secondary and tertiary effects were simulated, JAM decisively provided evidence for the existence of three signals (Table 1).

**Table 1:** Comparison of Bayes factors for multi‐SNP models when one and three signals were simulated

Simulation model	⩾1 SNP	⩾2 SNPs	⩾3 SNPs
Single SNP model	9.7*e*8	0.70	1.38
Multi‐SNP model	4.8*e*7	1.9*e*4	2.1*e*4

Notes

Large Bayes factors for more than and equal to two and three SNPs provide strong evidence for multi‐SNP models, whereas Bayes factors less than three are generally considered non‐statistically significant [Raftery, 1995]. Bayes factors are averages over 200 replicates.

All findings described in this section were equivalent across a range of alternative prior setups for the Bayesian models, including use of *g*‐priors vs. independence priors on the effects, different inverse‐gamma choices and other (larger) choices for the beta‐binomial parameter $b_{\omega }$. To explore how much performance we might lose through use of external correlation data and re‐construction of z from effect estimates, we repeated the JAM analyses for both scenarios, providing the true correlation structure used in the simulation model, and the actual group means z. Encouragingly, performance was very similar (supplementary Fig. S4), suggesting the approximations used in JAM are robust.

### Case Study

The MAGIC consortium have published results from a large‐scale GWAS meta‐analysis for glycemic and metabolic traits, involving more than 15,000 non‐diabetic individuals across nine cohorts. As is typical for consortium GWAS, only marginal SNP effects have been reported. As a case study, we re‐analyse MAGIC results corresponding to several loci highlighted for association with 2‐hr glucose levels after oral stimulation (a measure of glucose tolerance) by Saxena et al. [2010] – see their paper for full details of the original study. JAM requires an external individual‐level genotype matrix to re‐construct the correlation structure underlying marginal association results. In this case, we used genotype information from the UK WTCCC 2, 674 controls (genotyped on the Affymetrix 500K chip). These data are available from the consortium on request [WTCCC, 2007]. Because the MAGIC cohorts are also of European descent (see http://www.magicinvestigators.org/cohorts/), we expect the UK WTCCC sample to provide reasonable LD estimates.

JAM is limited to analysing SNPs available in the external reference data. Hence, our primary focus was on two of the top MAGIC loci for which the reported index was directly genotyped and available in the WTCCC (*TCF7L2* and *ADCY5*). However, we extended our case study to consider *GCKR* and *VPS13C*, two other top MAGIC loci for which the reported index was not directly genotyped in the WTCCC, but for which a strong tag was available (rs780094 with *D*' 0.96, and rs1436958 with *D*' 0.98, respectively). These tags were strong enough to capture most of the original signal, i.e. marginal *p*‐values reported by MAGIC were of a similar magnitude to the index. We applied JAM to the LD block surrounding the reported signal within each of these loci (see heatmaps in Supplementary Fig. S5). To avoid acute co‐linearity issues, we pruned SNPs to a maximum pairwise correlation of $r^{2}=0.95$ using PLINK [Purcell et al., 2007]. Furthermore, we pruned SNPs with MAF lower than 5%. For some SNPs the allele coding in MAGIC relative to WTCCC was ambiguous – in these cases, we inferred the correct coding according to the pattern of effects at correlated SNPs for which coding was certain.

Before running JAM, we analysed these regions using Yang et al's COJO algorithm, with the same WTCCC control genotypes as reference data. We found that the integrated stepwise procedure got stuck at the top marginal association for each gene. Likewise, PICS returned the index for each region as the single estimated causal SNP. Therefore, top hits from COJO and PICS were identical to those from the marginal *p*‐values.

**Figure 4:**
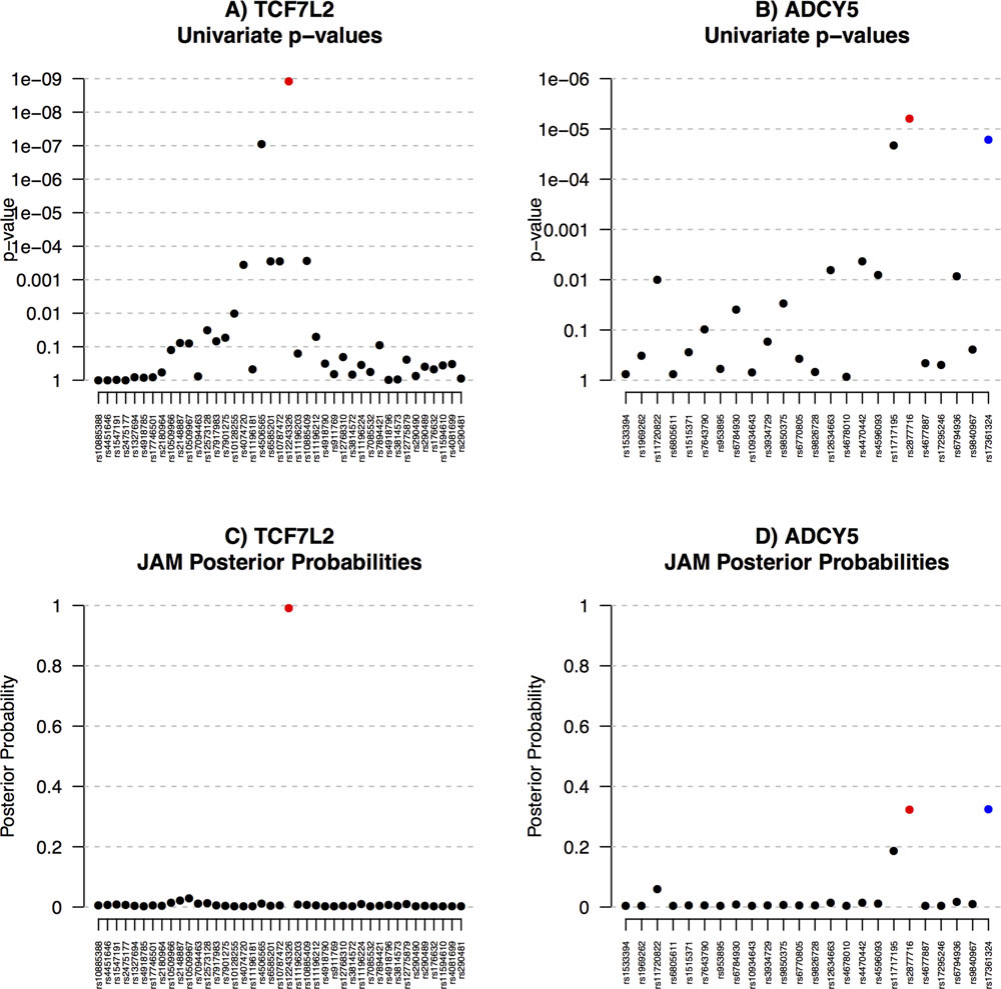
Application of JAM to marginal results reported by MAGIC for two of the top loci associated with 2‐hr fasting glucose. Two‐hour glucose levels after oral stimulation are a measure of glucose tolerance. Panels (A) and (B) display marginal one‐at‐a‐time *p*‐values, (C) and (D) display multivariate adjusted posterior probabilities as inferred by JAM. The MAGIC index SNPs are indicated in red. For *ADCY*, an additional SNP was highlighted by JAM – this is indicated in blue.

**Figure 5:**
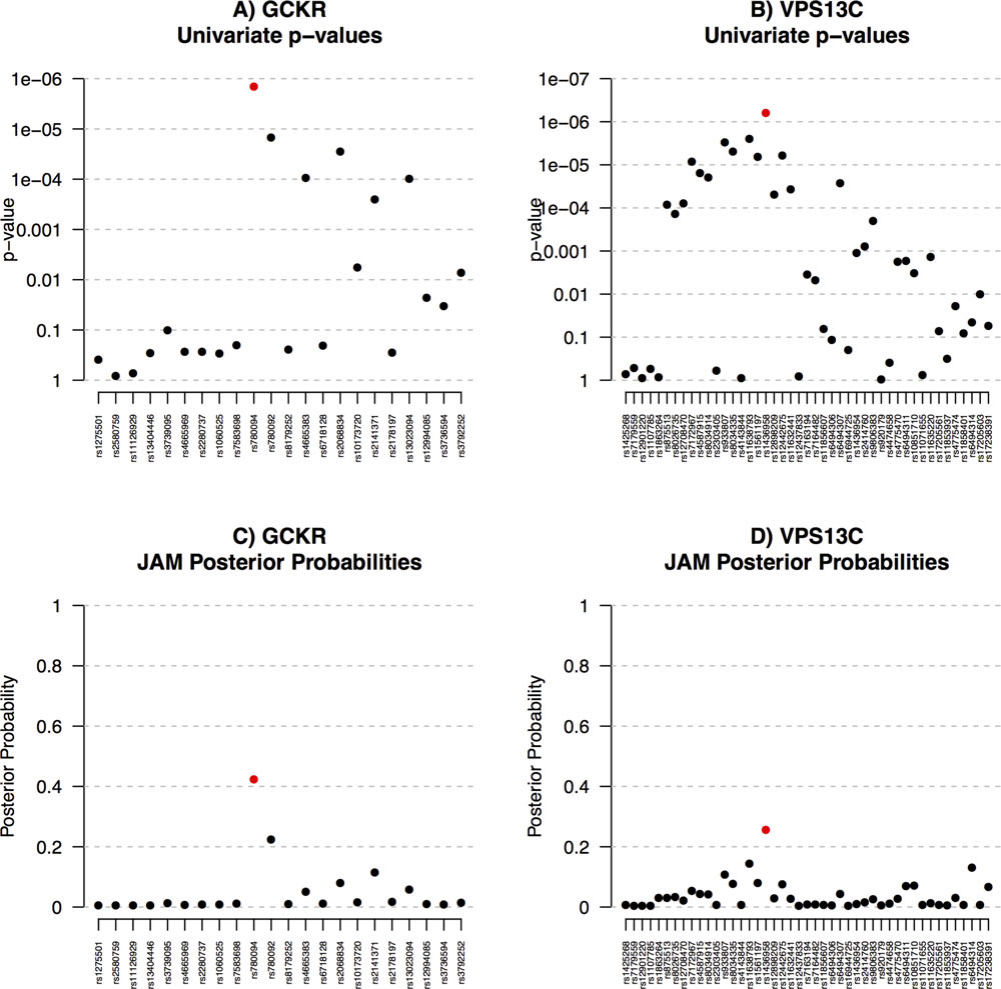
Application of JAM to marginal results reported by MAGIC for two of the top loci associated with 2‐hr fasting glucose, for which the MAGIC index SNP is represented by a tag. For *GCKR*, our tag SNP had *D*' 0.96 with the MAGIC index, and for *VPS13C*, our tag was in LD at *D*' 0.98. Both tags were the top SNPs. Panels (A) and (B) display marginal one‐at‐a‐time *p*‐values, (C) and (D) display multivariate adjusted posterior probabilities as inferred by JAM. For both genes JAM found no evidence for more than a single effect, although there was uncertainty around the location.

Next, we analysed the four regions simultaneously with JAM, using the same prior settings as described in the methods. We ran the stochastic model search for two million iterations (41 sec) which resulted in equivalent posterior probabilities across different MCMC chains, and compared to those inferred via enumeration of all models within each region of up to five SNPs, indicating convergence. The variable selection trace plot also displayed good mixing (see supplementary Fig. S9). For comparison, we also calculated the univariate APPs – see Figures 4 and 5. Through multivariate LD adjustment, JAM was able to rule out many marginal associations for each of these four loci. The APPs simply placed most weight on the top marginal SNP in each region and so produced identical top hits to the marginal *p*‐values, which is as to be expected as this is also a univariate method. For *TCF7L2*, JAM also confidently placed nearly all the posterior weight on a single SNP, re‐affirming the reported MAGIC index. For *ADCY5*, however, JAM gave equal posterior weights to the reported index (rs2877716) and another SNP, rs17361324. Given the correlation structure in the WTCCC data, JAM finds both SNPs equal candidates to explain the pattern of marginal effects over the locus (rs17361324 and rs2877716 are correlated at $D^{\prime }=96\%$). If two SNPs are correlated at a high enough degree, conditional modelling will be unable to delineate their effects, and splitting the posterior evidence correctly reflects that either SNP might best represent the signal. The spread of JAM's posterior weight over rs17361324 and rs2877716 was robust to a range of sensitivity analyses, including bootstrapping the WTCCC genotype rows to perturb the correlation estimates (see supplementary Fig. S6). Therefore, our LD‐aware multivariate re‐analysis suggests rs17361324 should also be considered for follow up with equal priority to the reported index. Interestingly, rs17361324 is more biologically plausible than the original index, as it is one of only three SNPs in the local region annotated with any known or predicted regulatory information. Specifically, this SNP is classified as ‘likely to affect binding’, and includes transcription factor binding and DNAase hypersensitivity.

For *GCKR* and *VPS13C*, where the reported MAGIC index SNPs were missing, JAM ascribed most posterior weight to our two tag SNPs, suggesting none of the other SNPs analysed here better capture the underlying signal. Finally, for all four regions, we estimated Bayes Factors for two or more underlying signals. For all genes there was no evidence for more than a single signal (Table 2). The top SNPs selected in each region, and their effect estimates are provided in Table 3. Findings were equivalent using different prior setups, and bootstrapping to perturb the WTCCC correlation estimates.

**Table 2:** Bayes factors in favour of multi‐SNP models among the MAGIC genes

Gene	⩾1 SNP	⩾2 SNPs
*TCF7L2*	133,739	<1
*ADCY5*	29	<1
*GCKR*	35	<1
*VPS13C*	161	<1

None of these genes demonstrated evidence for more than a single effect.

**Table 3:** Top SNPs selected by JAM in an application to four top loci reported by MAGIC for association with two hour fasting glucose

					MAGIC		JAM			
SNP	Gene	EA	NEA	MAF	Effect	*P*‐value	Effect	95% CrI	Post‐prob	Bayes factor
rs12243326	*TCF7L2*	T	C	0.24	−0.13	$1.2\times 10^{-9}$	−0.13	(−0.17, −0.09)	0.99	1,207
rs2877716	*ADCY5*	T	C	0.25	−0.10	$6.3\times 10^{-6}$	−0.10	(−0.16, −0.04)	0.32	4.78
rs17361324	*ADCY5*	T	C	0.26	−0.10	$1.6\times 10^{-5}$	−0.10	(−0.16, −0.04)	0.32	4.81
rs780094	*GCKR*	T	C	0.39	0.09	$1.4\times 10^{-6}$	0.09	(0.04, 0.14)	0.42	7.37
rs1436958	*VPS13C*	T	G	0.42	0.09	$6.2\times 10^{-7}$	0.09	(0.05, 0.14)	0.26	3.45

Notes

Two‐hour glucose levels after oral stimulation are a measure of glucose tolerance. These SNPs have been selected from a joint multivariate analysis of marginal results across each region. Marginal effects and *p*‐values as reported by MAGIC are also included for each SNP; there was no evidence for a multi‐SNP model in any region. Note that for *GCKR* and *VPS13C*, the MAGIC index was represented by a tag with *D*' 0.96 and 0.98, respectively. EA, effect allele; NEA, non‐effect allele.

## Discussion

We present a novel and scalable model selection framework to infer evidence for multivariate SNP associations from marginal effect estimates, accounting for correlation structure from a reference panel. The SNP or subset of SNPs which best explains the pattern of marginal effects across all SNPs in a region is highlighted via an integrated sparse Bayesian regression framework with variable selection. JAM proceeds by enumerating all models including up to three causal SNPs per region, but we have also implemented a more general version using Reversible Jump MCMC for when the number of causal SNPs may be larger or is unknown. In a series of realistic simulation studies, involving multi‐SNP effects and including an application to 10,000 SNPs, signal and tag SNPs were identified with greater specificity than an alternative multivariate stepwise selection procedure COJO. Furthermore, we demonstrate equivalent performance of JAM to other sparse regression summary statistics methods (namely CAVIARBF and FINEMAP) in a single region setting where all can be applied. We also present a real data application to published results from MAGIC – a GWAS meta‐analysis of more than 15,000 people, in which we re‐analyse several genomic regions associated with 2‐hr fasting glucose. Although JAM did not find evidence for multi‐SNP models, our case study highlights a subtler advantage of multivariate modelling; correctly reflecting model uncertainty in the presence of strong LD. For *ADCY5* this led to another SNP being highlighted for follow‐up with equal priority to the reported index and which is, in fact, more biologically plausible.

We build upon work by Verzilli et al. [2008], in which the multivariate likelihood of marginal statistics was formally derived as part of a sophisticated Bayesian meta‐analysis model. First, we invoke a Cholesky decomposition to transform the marginal associations into a vector of independently distributed statistics, thereby simplifying the likelihood expression. Second, we propose use of a conjugate *g*‐prior on the SNP effects, such that all parameters can be analytically integrated out. Consequently, the computational cost is dramatically reduced. The primary difference between JAM and other summary statistics sparse Bayesian regression methods, such as CAVIARBF and FINEMAP, is that JAM has been implemented for application to multiple regions, and that JAM incorporates a full MCMC model search algorithm. There are, however, some conceptual advantages too. JAM's formulation integrates over uncertainty in the residual error, as would be done in a traditional regression analysis, rather than fix it at an assumed value. Furthermore, we use a *g*‐prior on the distribution of effects, which has been shown to help when covariates are highly correlated [Bottolo and Richardson, 2010]. Despite these conceptual differences CAVIARBF and FINEMAP demonstrated equivalent performance when compared to JAM in a single region setting. If CAVIARBF and FINEMAP were extended for application to multiple regions in the same way, the resulting performance may be very similar to JAM's.

JAM relies on the assumption that the reference genotype data are taken from the same population, such that LD estimates are unbiased. A simulation experiment showed no difference in results using an independent sample from the same underlying population, suggesting the size of our WTCCC reference sample, *n*=2,674, is sufficiently large to produce accurate correlation estimates. This is consistent with a simulation study reported by Yang et al. [2012], recommending a minimum reference sample size of 2,000. A limitation of JAM is that for every LD block analysed, a full rank reference genotype matrix is required. Therefore, each LD block cannot be larger than the reference sample size, and are further limited in practice by the level of LD. Naturally we recommend using the largest comparable reference data to minimise error in the LD estimates and allow analysis of all important LD blocks in their entirety. For our case study, the WTCCC sample was sufficient when pruning at $r^{2}=0.95$, providing us with full rank genotype matrices for each candidate region analysed. With the impending availability of the UK10K, a publicly available resource of 10,000 people genotyped at high density, these issues should no longer effect analyses of individuals of European descent and will permit application of JAM to larger LD blocks with correlations stronger than $r^{2}=0.95$, allowing finer mapping than presented in our case study.

An alternative and complementary line of research for re‐prioritising marginal associations involves integrative analysis with the increasing wealth of functional genomic annotation information available from consortia such as ENCODE [Flicek et al., 2014]. A simple illustration clearly demonstrates the appeal of such approaches. Consider two variants in perfect LD, but only one of which causally effects a trait of interest. Both variants receive identical marginal measures of association, however, post hoc consideration of functional annotation – e.g. if the true variant impacts the trait through a non‐synonymous change in the corresponding protein – may clearly point to which has the true effect. Notable methods include that of Quintana and Conti [2013] for multivariate modelling of IPD and of Pickrell [2014] and Kichaev et al. [2014] for summary data, in which a hierarchical Bayesian model to learn the relative importance of different annotation features. Such approaches show promise in aiding variant prioritisation when (reliable) annotation information is available for causal SNPs. It would be natural going forward to borrow from these ideas and extend JAM, by specifying SNP‐specific beta‐binomial prior parameters, weighted to reflect annotation information.

A notable limitation is that JAM is not currently designed for the analysis of marginal odds ratios, i.e. summary effect estimates from a case‐control study. This is due to the derivation in terms of a Gaussian linear regression model for continuous data. Pirinen et al. [2013] have explored, in the genetic setting, a method for inferring approximate odds ratios under a linear regression model in which the residual error term is fixed to a function of the case/control sampling fraction. They found the approximation works well in the typical GWAS context of small effect sizes [Pirinen et al., 2013]. Application of JAM with summary odds ratios may be possible under this transformation, as has been demonstrated in related fixed residual frameworks [Benner et al., 2015; Chen et al., 2015]. We leave exploration of this adaption, and whether inference can be improved by using JAM's fully Bayesian framework to target the approximate residual function with an informative prior, rather than a fixed value, to future work. In previous work, Newcombe et al. [2009] derived the true likelihood of summary odds ratios in terms of their multivariate adjusted effects and haplotype information from a reference sample, under an assumption of additive effects. The approach was implemented within a Bayesian variable selection framework, but was not designed to scale beyond the analysis of tens of SNPs. In future work, we also plan to explore an extension of JAM based on this same haplotype formulation, which would allow formal inference of multivariate odds ratios from case‐control summary data.

JAM's Reversible Jump stochastic search algorithm, which avoids an assumption on the maximum number of causal SNPs, demonstrated equal performance to inference obtained by exhaustively evaluating all possible configurations of up to three causal SNPs in each region but took longer to run (3 hr for 10,000 SNPs). We plan to build on the current simple add/delete/swap algorithm to implement a more sophisticated model sampling scheme, e.g. using ideas from population MCMC in which parallel chains are run at different ‘temperatures’, swapping information to overcome local optima in the model space [Bottolo and Richardson, 2010]. This should lead to an improved algorithm that reliably covers the model space in less iterations, thereby improving speed. For a more detailed exploration of when the benefits of a stochastic search outweigh exhaustive enumeration, we refer readers to Benner et al.'s [2015] article describing the FINEMAP algorithm. We reserve a more detailed exploration of this trade‐off in the context of multiple regions and JAM to further work.

To facilitate ease of use, we have incorporated the JAM algorithm into our existing fully documented R package for Bayesian model selection, which is freely available to download (along with a vignette specifically for JAM) via github https://github.com/pjnewcombe/R2BGLiMS.

## Supporting information

Disclaimer: Supplementary materials have been peer‐reviewed but not copyedited.

**Figure S1**: Performance of JAM under the null, for a range of beta‐binomial prior choices. For each simulated SNP, average posterior probabilities over 200 replicates are given. Ideally, since no signal exists, all averages should be near 0.
**Figure S2**: JAM run times for posterior model inference by enumeration up to dimension 3 for different numbers of SNPs. SNPs and analyses were decomposed into the same LD blocks used in the simulations. Run times are plotted for an analysis of 132 SNPs over 4 blocks, 1000 SNPs over 32 blocks, 5000 SNPs over 152 blocks and 10000 SNPs over 304 blocks. Analyses were run on an Intel Xeon E5‐2640 2.50GHz processor.
**Figure S3**: Comparison of ranking performance by JAM against various other strategies when data were simulated under single SNP models for 15,356 individuals (the total size of the MAGIC consortium). Ranking performance is measured in terms of positive predictive value (PPV), the proportion of true signal SNPs in the selection (solid lines, left y‐axis), and power/sensitivity, the proportion of all simulated signals included (dashed lines, right y‐axis). For each method, the average PPV and sensitivity estimates consist of points for each SNP rank, which we have joined with lines to ease the visual comparison. 132 SNPs were simulated across 4 regions, each of which had a single effect. For LD estimation, JAM was provided with an independently simulated reference dataset of 2,674, the size of the WTCCC control sample. Estimates are averaged over 200 simulation replicates. A vertical grey line highlights the rank equal to the number of true signals, where PPV and sensitivity by definition intersect. IPD: Individual Patient Data, ABF: Wakefield's Approximate Bayes Factor.
**Figure S4**: Comparison of JAM performance between use of the true correlation structure and true trait group means vs correlation structure from an independent sample and trait group means reconstructed from marginal effect estimates. Ranking performance is measured in terms of positive predictive value (PPV), the proportion of true signal SNPs in the selection (solid lines, left y‐axis), and power/sensitivity, the proportion of all simulated signals included (dashed lines, right y‐axis). For each method, the average PPV and sensitivity estimates consist of points for each SNP rank, which we have joined with lines to ease the visual comparison. Panel A) corresponds to a fine‐mapping scenario including 132 SNPs across 4 regions, and panel B) corresponds to a higher dimensional setting in which 40 effects were simulated among 10,000 SNPs. Estimates are averaged over 200 simulation replicates. A vertical grey line highlights the rank equal to the number of true signals, where PPV and sensitivity by de
nition intersect. IPD: Individual Patient Data; the full IPD multivariate analysis is presented to demonstrate ‘optimal’ performance.
**Figure S5**: LD blocks analysed for each of the four genes re‐analysed from the MAGIC consortium results. Pairwise pearson *r* ^2^ is plotted for each combination of SNPs (black = 1, white = 0). The red boxes indicate the correlation block (around the MAGIC reported index) analysed for each gene in our case study, and the red dotted line highlights the index SNP (or our tag in the case of GCKR and VPS13C). The location of additional effects in our multi‐SNP simulation studies are indicated in blue.
**Figure S6**: Sensitivity analysis for ADCY5. The reference correlation structure from WTCCC was perturbed by bootstrapping rows (with replacement) 10 times.
**Figure S7**: Example trace plot of selection indicators provided by JAM in an analysis of simulated summary statistics from 15,356 individuals (the total size of the MAGIC consortium) for 132 SNPs over 4 regions. Multi‐SNP signals (3 effects) were simulated in each region. Predictors are ordered horizontally, and posterior samples from JAM are ordered vertically from bottom to top. For each SNP, inclusion at the particular iteration is denoted in black, and exclusion is denoted in white. This plot helps to visualise the mixing patterns of the selection indicators and if variables seem to stick, i.e. do not come in and out in a fairly regular manner. JAM was run for 2 million iterations.
**Figure S8**: Example trace plot of selection indicators provided by JAM in an analysis of simulated summary statistics from 15,356 individuals (the total size of the MAGIC consortium) for 10,000 SNPs over 304 regions. Multi‐SNP signals (3 effects) were simu‐ lated in four of the regions. Predictors are ordered horizontally, and posterior samples from JAM are ordered vertically from bottom to top. For each SNP, inclusion at the particular iteration is denoted in black, and exclusion is denoted in white. This plot helps to visualise the mixing patterns of the selection indicators and if variables seem to stick, i.e. do not come in and out in a fairly regular manner. JAM was run for 20 million iterations.
**Figure S9**: Trace plot of selection indicators provided by JAM in an analysis of summary statistics published by the MAGIC consortium for 132 SNPs over four regions analysed in 15,356 individuals for association with glucose 2 hours after oral stimulation. SNPs are ordered horizontally, and posterior samples from JAM are ordered vertically from bottom to top. For each SNP, inclusion at the particular iteration is denoted in black, and exclusion is denoted in white. This plot helps to visualise the mixing patterns of the selection indicators and if variables seem to stick, i.e. do not come in and out in a fairly regular manner. JAM was run for 2 million iterations. Sticking can be seen during the first million iterations, but note that these iterations (the first half) were discarded as part of the burn‐in
