# Supplementary material for: JAM: A Scalable Bayesian Framework for Joint Analysis of Marginal SNP Effects
Source: Genet Epidemiol. 2016 Mar 29;40(3):188–201. doi: 10.1002/gepi.21953 (PMC4817278; doi:10.1002/gepi.21953)
Supplement: Supplementary file 1 — Figure S1: Performance of JAM under the null, for a range of beta‐binomial prior choices. For each simulated SNP, average posterior probabilities over 200 replicates are given. Ideally, since no signal exists, all averages should be near 0. Figure S2: JAM run times for posterior model inference by enumeration up to dimension 3 for different numbers of SNPs. SNPs and analyses were decomposed into the same LD blocks used in the simulations. Run times are plotted for an analysis of 132 SNPs over 4 blocks, 1000 SNPs over 32 blocks, 5000 SNPs over 152 blocks and 10000 SNPs over 304 blocks. Analyses were run on an Intel Xeon E5‐2640 2.50GHz processor. Figure S3: Comparison of ranking performance by JAM against various other strategies when data were simulated under single SNP models for 15,356 individuals (the total size of the MAGIC consortium). Ranking performance is measured in terms of positive predictive value (PPV), the proportion of true signal SNPs in the selection (solid lines, left y‐axis), and power/sensitivity, the proportion of all simulated signals included (dashed lines, right y‐axis). For each method, the average PPV and sensitivity estimates consist of points for each SNP rank, which we have joined with lines to ease the visual comparison. 132 SNPs were simulated across 4 regions, each of which had a single effect. For LD estimation, JAM was provided with an independently simulated reference dataset of 2,674, the size of the WTCCC control sample. Estimates are averaged over 200 simulation replicates. A vertical grey line highlights the rank equal to the number of true signals, where PPV and sensitivity by definition intersect. IPD: Individual Patient Data, ABF: Wakefield's Approximate Bayes Factor. Figure S4: Comparison of JAM performance between use of the true correlation structure and true trait group means vs correlation structure from an independent sample and trait group means reconstructed from marginal effect estimates. Ranking performance is meas [file GEPI-40-188-s001.pdf]

Supplemental Material for 'JAM: A scalable Bayesian framework for joint analysis of marginal SNP effects'

P. J . Newcombe[1], D. V. Conti[2,*], and S. Richardson[1,*]

[1]MRC Biostatistics Unit, Cambridge, UK
[2]Division of Biostatistics, Department of Preventive Medicine, Zilkha Neurogenetic Institute, University of Southern California, US
[*]*These authors contributed equally to this work.*

1 Supplementary Methods

Constructing $\boldsymbol{z}$ from univariate effect estimates

More often than not, the summary data available for each marker m will consist only of a univariate effect estimate, $\hat{\beta}_m$. However, we can construct estimates of the genotype group means, $\bar{y}_{mg}$, and counts, n_{mg}, required to define $\boldsymbol{z}$ as follows. First, using an estimate of marker m's minor allele frequency, $\hat{p}_m$, taken either from the original study or an external source such as the 1000 genomes project, the group counts, n_{mg}, are straight forward to estimate if we assume the marker has reached Hardy Weinberg Equilibrium (HWE):

$$\hat{n}_{m0} = (1 - \hat{p}_m)^2$$
$$\hat{n}_{m1} = 2\hat{p}(1 - \hat{p}_m)$$
$$\hat{n}_{m2} = \hat{p}_m^2$$

Next, define $\bar{y}_{Pop}$ as the population mean value of y. Then, for all m, this may be written as a weighted average of the group means, $\bar{y}_{mg}$, weighted by the true group counts;

$$\bar{y}_{Pop} = \frac{n_{0m}\bar{y}_{0m} + n_{1m}\bar{y}_{1m} + n_{2m}\bar{y}_{2m}}{n_{0m} + n_{1m} + n_{2m}}$$

As described earlier, for simplicity, we model the mean-centred phenotype, y, to avoid fitting an intercept term. Therefore we have:

$$0 = \frac{n_{0m}\bar{y}_{0m} + n_{1m}\bar{y}_{1m} + n_{2m}\bar{y}_{2m}}{n_{0m} + n_{1m} + n_{2m}} \tag{1}$$

If the assumptions underlying the linear regression model from which $\hat{\beta}_m$ was estimated hold perfectly, we also have;

$$\bar{y}_{1m} = \bar{y}_{0m} + \hat{\beta}_m \tag{2}$$

and

$$\bar{y}_{2m} = \bar{y}_{0m} + 2\hat{\beta}_m \tag{3}$$

Since we can estimate the group counts, we have a system of three simultaneous equations (1), (2), and (3) in three unknowns, which may thus be solved to obtain estimates of the three group means. Hence, substituting in the group count estimates, $\hat{n}_{mg}$ from above, and then substituting equations (2) and (3) into (1) we obtain the following approximation for the wildtype mean;

$$0 \approx \frac{\hat{n}_{m0}\bar{y}_{m0} + \hat{n}_{m1}(\bar{y}_{m0} + \hat{\beta}_m) + \hat{n}_{m2}(\bar{y}_{m0} + 2\hat{\beta}_m)}{\hat{n}_{m0} + \hat{n}_{m1} + \hat{n}_{m2}}$$

therefore:

$$\hat{\bar{y}}_{m0} = -\frac{\hat{n}_{m1}\hat{\beta}_m + 2\hat{n}_{m2}\hat{\beta}_m}{\hat{n}_{m0} + \hat{n}_{m1} + \hat{n}_{m2}}$$

Approximations for $\bar{y}_{m1}$ and $\bar{y}_{m2}$ then follow from equations (2) and (3):

$$\hat{\bar{y}}_{m1} = \hat{\bar{y}}_{m0} + \hat{\beta}_m$$

$$\hat{\hat{y}}_{m2} = \hat{\hat{y}}_{m0} + 2\hat{\beta}_m$$

We may now construct an approximation for $\boldsymbol{z}$ from $\hat{n}_{mg}$ and $\hat{\hat{y}}_{mg}$, using equation (2). Therefore, our framework may be used to infer joint effect estimates from univariate effect estimates, providing a full IPD genotype matrix is available from an external population (note that this genotype matrix can be used to derive MAF estimates p_m, as well as the plug-in estimate for $\boldsymbol{X}'\boldsymbol{X}$).

Construction of plug-in estimate for $\boldsymbol{X}'\boldsymbol{X}$

We follow the method described by Yang and Visscher[2], to construct a plug-in estimate for $\boldsymbol{X}'\boldsymbol{X}$, the unobserved $P \times P$ genotype variance-covariance matrix. Define as $\boldsymbol{W}$ the genotype matrix from an external population (such as the 1,000 genonomes project). For consistency with our intercept free formulation, genotypes for SNP m will be centred in $\boldsymbol{W}$ such that for all individuals i, $w_{i,m} = -2p_m, 1-2p_m$ or $2p_m$ where p_m is the frequency of SNP m in the external population. If the external population is of identical size to the analysis population, we could simply approximate $\boldsymbol{X}'\boldsymbol{X}$ with $\boldsymbol{W}'\boldsymbol{W}$. However, if the external population is of a different size (as will nearly always be the case) we must scale the variance and co-variances accordingly. First define $\boldsymbol{D}_W$ as the diagonal matrix of $\boldsymbol{W}'\boldsymbol{W}$, such that $D_{W(m)}$, the mth diagonal entry corresponding to marker m, is easily obtained from $\boldsymbol{W}$ as $\sum_i w_{i,m}^2$. Similarly, define $\boldsymbol{D}$ as the diagonal matrix of $\boldsymbol{X}'\boldsymbol{X}$. We have therefore not observed $\boldsymbol{D}$, however, assuming HWE, it is straight forward to show that the mth entry may approximated according to MAF p_m from the external data as:

$$D_m \approx 2p_m(1-2p_m)n$$

Note that this approximation accounts for the mean centred genotype coding; the expression would be different expression if this was not the case. We may now approximate $\boldsymbol{X}'\boldsymbol{X}$ with $\boldsymbol{B}$, where;

$$B_{j,k} = \sqrt{\frac{D_j D_k}{D_{W(j)} D_{W(k)}}} \sum_i w_{i,j} w_{i,k}$$

Alternatively, in matrix form

$$\boldsymbol{X}'\boldsymbol{X} \approx \boldsymbol{B} = \boldsymbol{D}^{1/2}\boldsymbol{D}_W^{-1/2}\boldsymbol{W}'\boldsymbol{W}\boldsymbol{D}_W^{-1/2}\boldsymbol{D}^{1/2}$$

Exploiting block independence to extend to high dimensional problems

The approximate inference approach described in the methods can be efficiently extended to large numbers of SNPs if the block independence decomposition described in the online methods is invoked (which will likely be necessary anyhow). We start by defining the approximate normalising constant corresponding to to an analysis of block b in isolation, considering models $\gamma_b \in \Gamma_b^*$ up to dimension 3:

$$\hat{C}_b = \sum_{\gamma_b \in \Gamma_b^*} p(\gamma_b) p(\gamma_b | \boldsymbol{L}_b^{-1} \boldsymbol{z}_b) \tag{4}$$

The normalising constant corresponding to the joint analysis of all blocks, considering all possible models up to dimension 3 within each block, may be written as:

$$\hat{C} = \sum_{\gamma_1 \in \Gamma_1^*} ... \sum_{\gamma_B \in \Gamma_B^*} p(\gamma_1, ..\gamma_B) p(\gamma_1, ..\gamma_B | \boldsymbol{L}_1^{-1} \boldsymbol{z}_1, ..\boldsymbol{L}_B^{-1} \boldsymbol{z}_B)$$

Under the block independence likelihood decomposition, and placing independent beta-binomial (a_ω, b_ω) priors on model dimension in each block this becomes:

$$\begin{aligned} \hat{C} &= \sum_{\gamma_1 \in \Gamma_1^*} ... \sum_{\gamma_B \in \Gamma_B^*} p(\gamma_1) p(\gamma_1 | \boldsymbol{L}_1^{-1} \boldsymbol{z}_1) ... p(\gamma_B) p(\gamma_B | \boldsymbol{L}_B^{-1} \boldsymbol{z}_B) \\ &= \prod_{b=1,..B} \hat{C}_b \end{aligned} \quad (5)$$

where each $\hat{C}_b$ is given by (4). If we are interested in a particular SNP or combination of SNPs in block b, γ_b say, the approximate posterior probability marginalised over all other blocks reduces to that which would be obtained from an analysis of the block in isolation:

$$\begin{aligned} p(\gamma_b) \approx\ & \hat{C}^{-1} p(\gamma_b) p(\gamma_b | \boldsymbol{L}_b^{-1} \boldsymbol{z}_b) \\ & \sum_{\gamma_1 \in \Gamma_1^*} ... \sum_{\gamma_{b-1} \in \Gamma_{b-1}^*} \sum_{\gamma_{b+1} \in \Gamma_{b+1}^*} ... \sum_{\gamma_B \in \Gamma_B^*} \\ & p(\gamma_1) p(\gamma_1 | \boldsymbol{L}_1^{-1} \boldsymbol{z}_1) ... p(\gamma_{b-1}) p(\gamma_{b-1} | \boldsymbol{L}_{b-1}^{-1} \boldsymbol{z}_{b-1}) p(\gamma_{b+1}) p(\gamma_{b+1} | \boldsymbol{L}_{b+1}^{-1} \boldsymbol{z}_{b+1}) ... p(\gamma_B) p(\gamma_B | \boldsymbol{L}_B^{-1} \boldsymbol{z}_B) \\ =\ & \hat{C}^{-1} p(\gamma_b) p(\gamma_b | \boldsymbol{L}_b^{-1} \boldsymbol{z}_b) \hat{C}_1 ... \hat{C}_{b-1} \hat{C}_{b+1} ... \hat{C}_B \\ =\ & \hat{C}_b^{-1} p(\gamma_b) p(\gamma_b | \boldsymbol{L}_b^{-1} \boldsymbol{z}_b) \end{aligned} \quad (6)$$

Therefore, invoking the block independence assumption and using independent but calibrated beta-binomial priors, the genome-wide relative importance of block specific SNPs or models may be estimated from an analysis of each block in isolation. Computationally speaking the genome-wide marginal scan amounts to no more than the total computation of analysing each block in isolation - that is, computation increases linearly with number SNPs. Specifically, the number of calculations, or evaluations of equation (9) and (10) in the online methods:

$$\sum_{b=1,..B} P_b$$

where P_b is the total number of models in the truncated model space, Γ_b^*, corresponding to block b.

Model fitting via Reversible Jump MCMC

Here we describe how, alternatively to enumeration of models up to dimension 3, a Reversible Jump MCMC scheme and be used to sample from the posterior $p(\gamma | \boldsymbol{z}_L)$ described in the online methods[1]. The Reversible Jump sampling scheme starts at an initial model, which we denote $\boldsymbol{\gamma}(0)$. To sample the next model, $\boldsymbol{\gamma}(1)$, we propose moving from the

current model to another model $\boldsymbol{\gamma}*$ using a proposal function $q(\boldsymbol{\gamma} * |\boldsymbol{\gamma})$. We then accept the proposed model as the next sample with probability equal to the Metropolis-Hastings ratio:

$$MHR = \frac{P(\boldsymbol{z}_L|\boldsymbol{\gamma}*)P(\boldsymbol{\gamma}*)}{P(\boldsymbol{z}_L|\gamma)P(\gamma)} \times \frac{q(\gamma|\boldsymbol{\gamma}*)}{q(\boldsymbol{\gamma} * |\boldsymbol{\gamma})}$$

where $P(\boldsymbol{z}_L|\boldsymbol{\gamma}*)$ is the likelihood described in equation (9) and $P(\boldsymbol{\gamma})$ is the beta-binomial model space prior defined in equation (10) of the online methods. Therefore the proposed model is accepted with a probability proportional to it's likelihood and prior. If the new model is accepted, the proposed sample is accepted as $\boldsymbol{\gamma}(1)$; otherwise, the sample remains equal to the current model, i.e., $\boldsymbol{\gamma}(1) = \boldsymbol{\gamma}(0)$. It can be shown that this produces a sequence of samples that converge to the required posterior distribution[1]. The algorithm is implemented in our software, and can optionally be used instead of the enumeration approach described in the methods.

2 Supplementary Figures

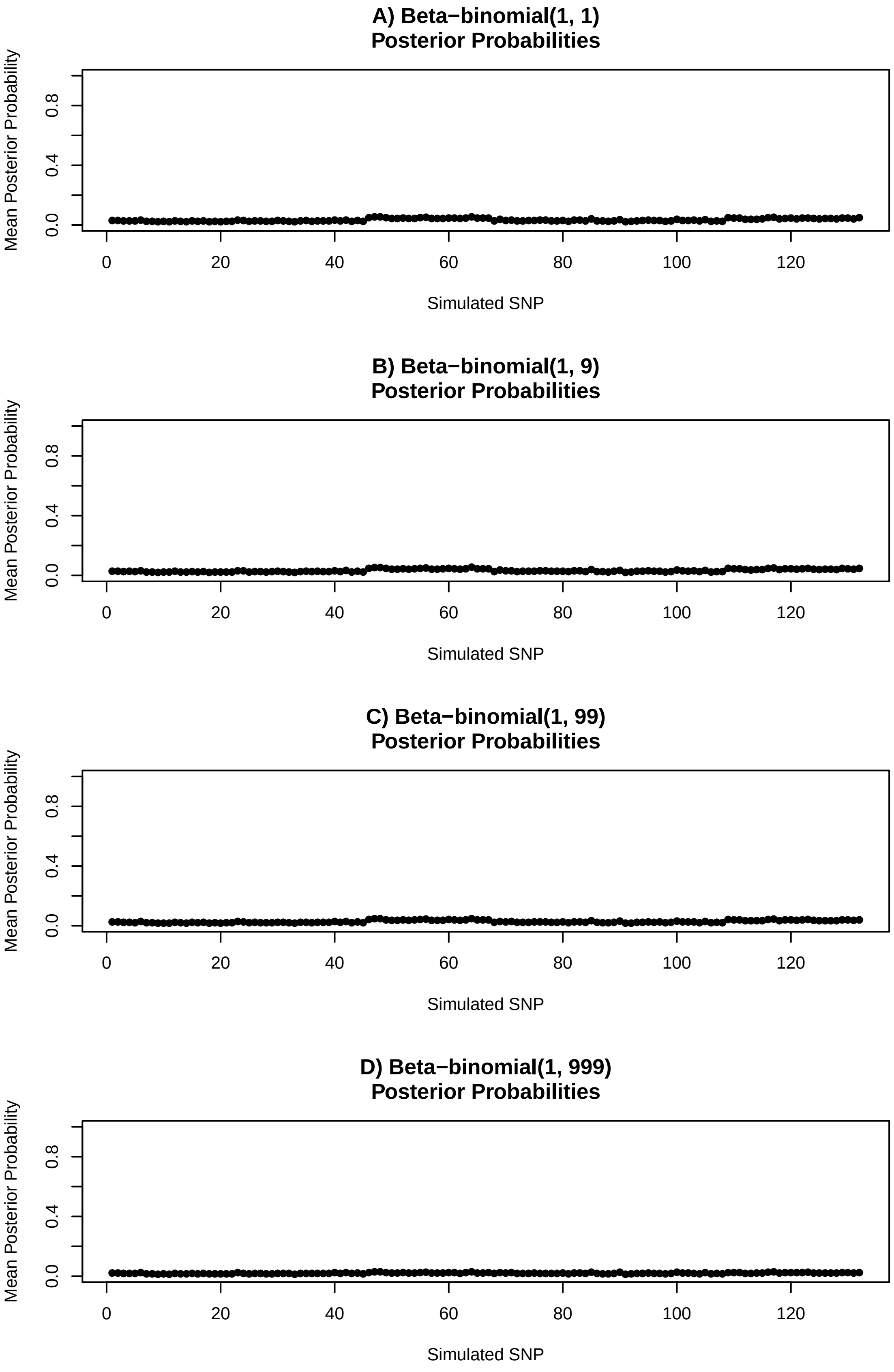

Figure S1: Performance of JAM under the null, for a range of beta-binomial prior choices. For each simulated SNP, average posterior probabilities over 200 replicates are given. Ideally, since no signal exists, all averages should be near 0.

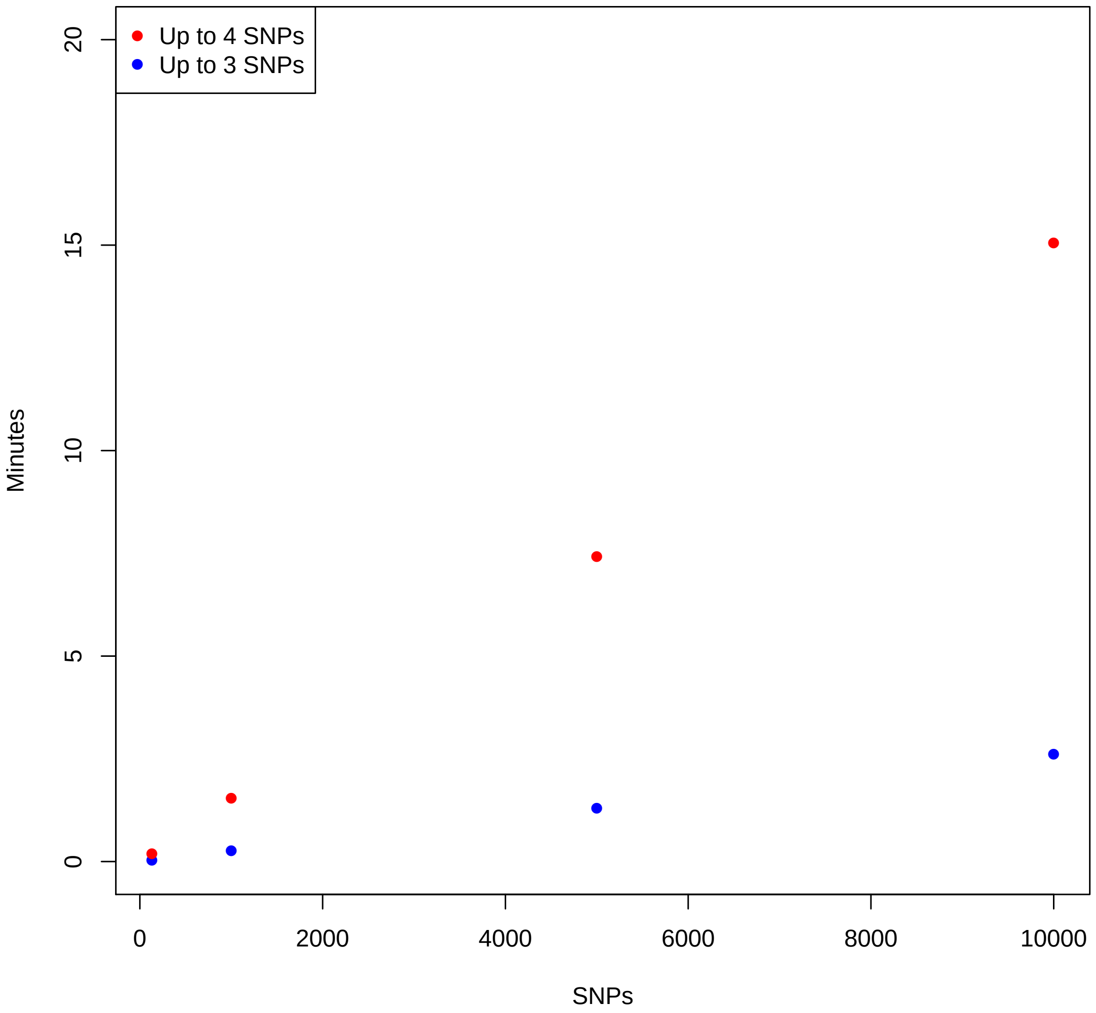

Figure S2: JAM run times for posterior model inference by enumeration up to dimension 3 for different numbers of SNPs. SNPs and analyses were decomposed into the same LD blocks used in the simulations. Run times are plotted for an analysis of 132 SNPs over 4 blocks, 1000 SNPs over 32 blocks, 5000 SNPs over 152 blocks and 10000 SNPs over 304 blocks. Analyses were run on an Intel Xeon E5-2640 2.50GHz processor.

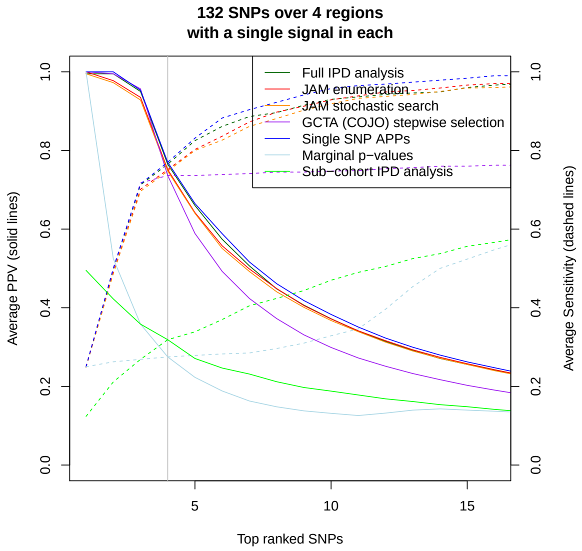

Figure S3: Comparison of ranking performance by JAM against various other strategies when data were simulated under single SNP models for 15,356 individuals (the total size of the MAGIC consortium). Ranking performance is measured in terms of positive predictive value (PPV), the proportion of true signal SNPs in the selection (solid lines, left y-axis), and power/sensitivity, the proportion of all simulated signals included (dashed lines, right y-axis). For each method, the average PPV and sensitivity estimates consist of points for each SNP rank, which we have joined with lines to ease the visual comparison. 132 SNPs were simulated across 4 regions, each of which had a single effect. For LD estimation, JAM was provided with an independently simulated reference dataset of 2,674, the size of the WTCCC control sample. Estimates are averaged over 200 simulation replicates. A vertical grey line highlights the rank equal to the number of true signals, where PPV and sensitivity by definition intersect. IPD: Individual Patient Data, ABF: Wakefield's Approximate Bayes Factor.

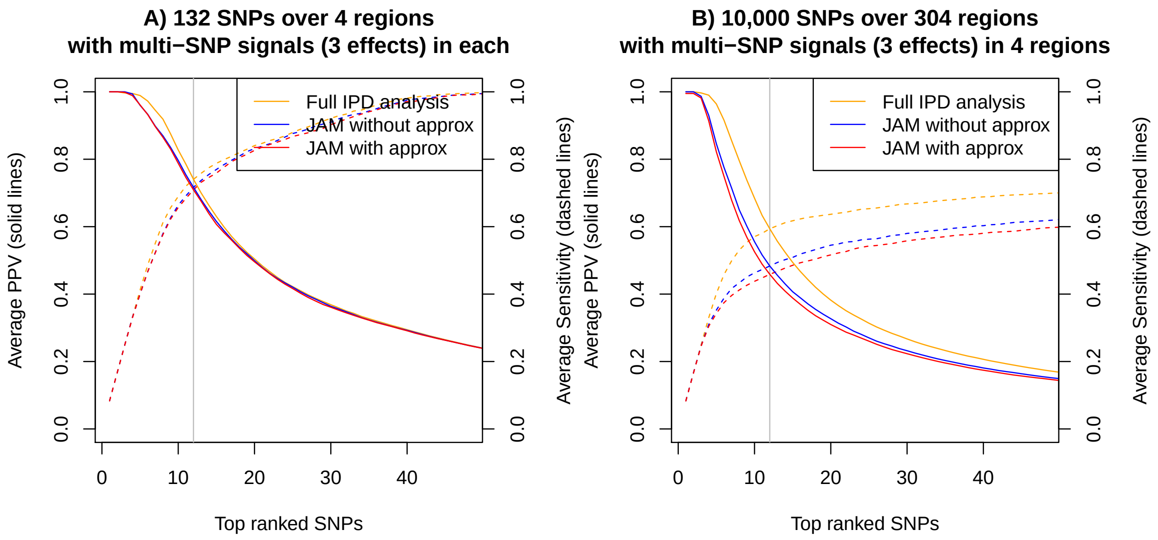

Figure S4: Comparison of JAM performance between use of the true correlation structure and true trait group means vs correlation structure from an independent sample and trait group means reconstructed from marginal effect estimates. Ranking performance is measured in terms of positive predictive value (PPV), the proportion of true signal SNPs in the selection (solid lines, left y-axis), and power/sensitivity, the proportion of all simulated signals included (dashed lines, right y-axis). For each method, the average PPV and sensitivity estimates consist of points for each SNP rank, which we have joined with lines to ease the visual comparison. Panel A) corresponds to a fine-mapping scenario including 132 SNPs across 4 regions, and panel B) corresponds to a higher dimensional setting in which 40 effects were simulated among 10,000 SNPs. Estimates are averaged over 200 simulation replicates. A vertical grey line highlights the rank equal to the number of true signals, where PPV and sensitivity by definition intersect. IPD: Individual Patient Data; the full IPD multivariate analysis is presented to demonstrate 'optimal' performance.

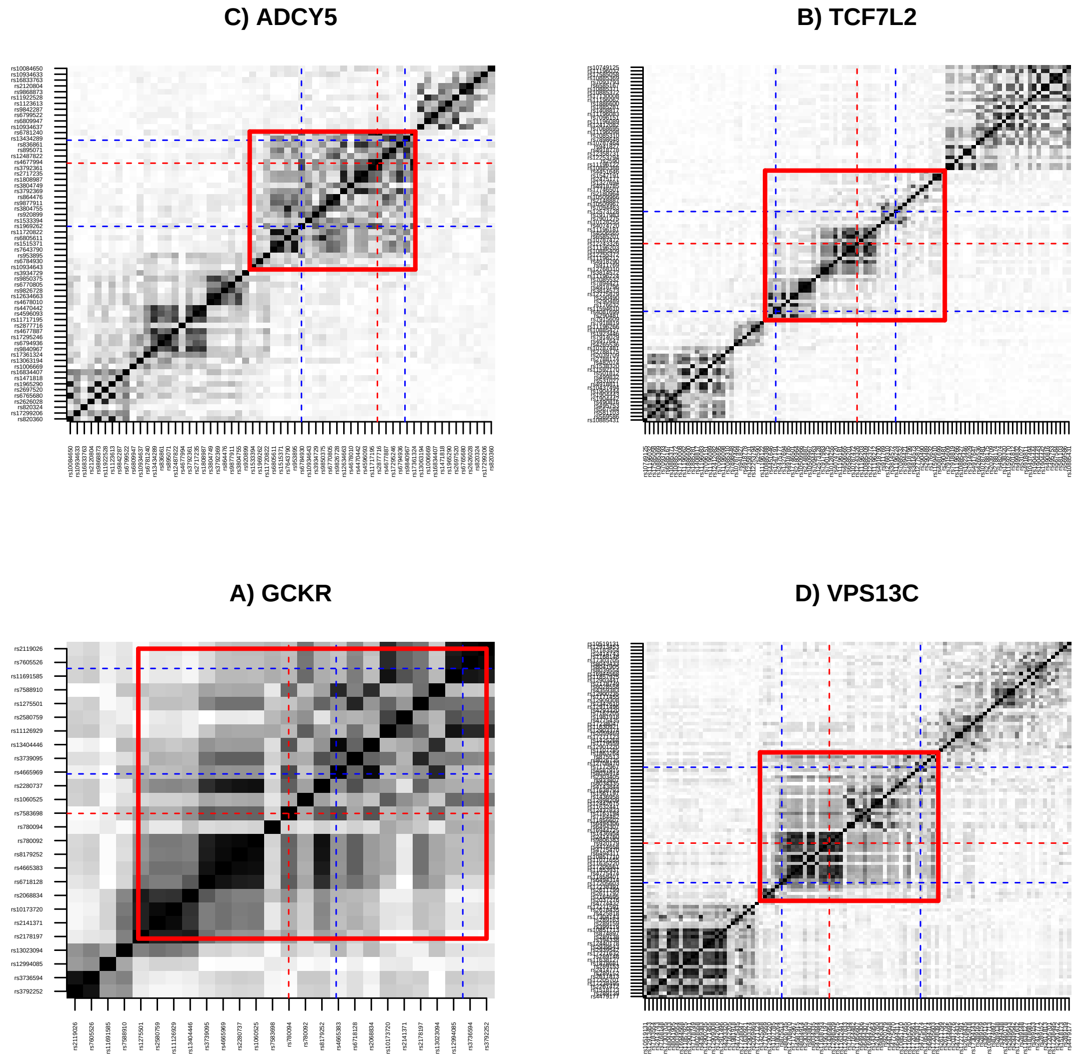

Figure S5: LD blocks analysed for each of the four genes re-analysed from the MAGIC consortium results. Pairwise pearson r^2 is plotted for each combination of SNPs (black = 1, white = 0). The red boxes indicate the correlation block (around the MAGIC reported index) analysed for each gene in our case study, and the red dotted line highlights the index SNP (or our tag in the case of *GCKR* and *VPS13C*). The location of additional effects in our multi-SNP simulation studies are indicated in blue.

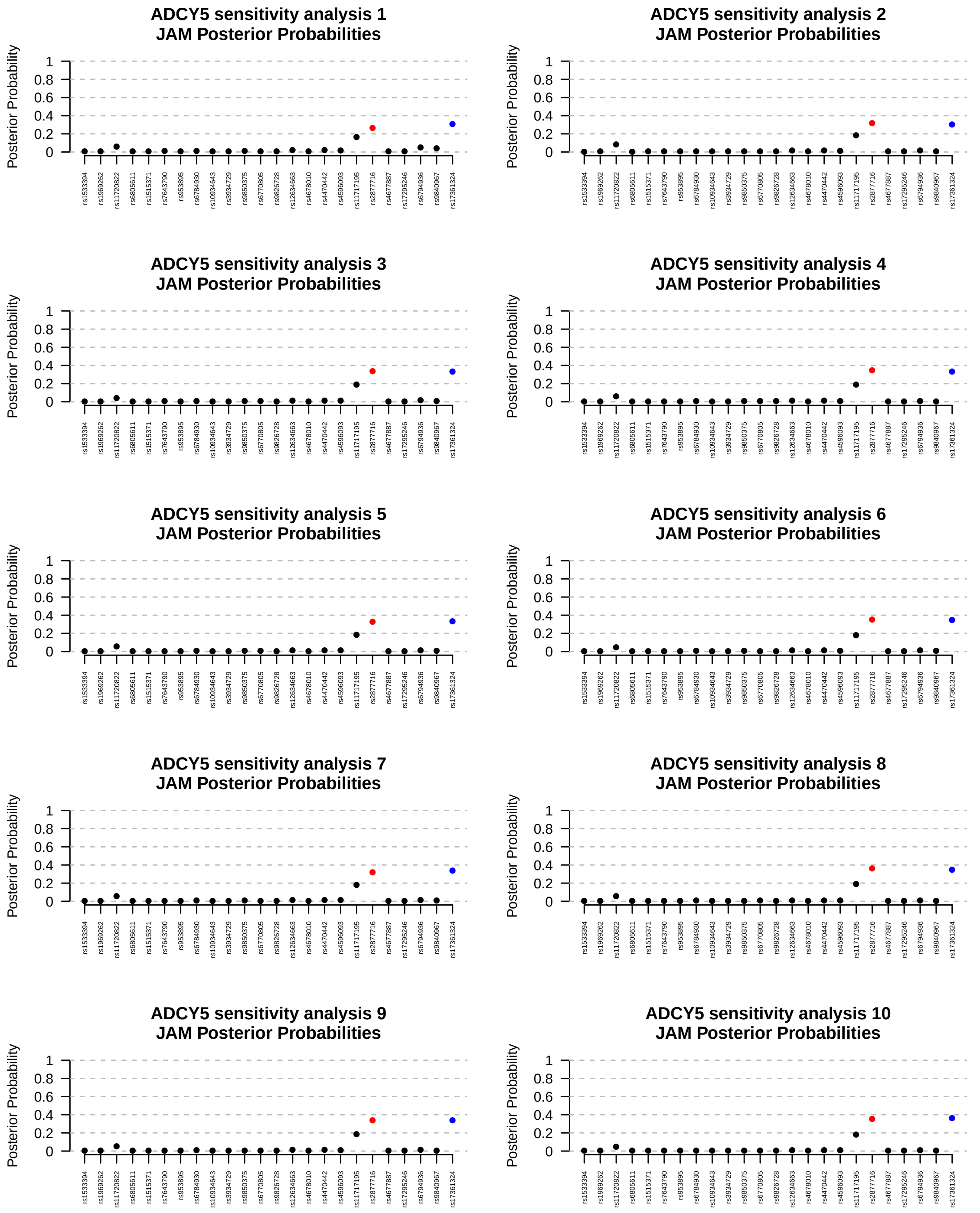

Figure S6: Sensitivity analysis for *ADCY5*. The reference correlation structure from WTCCC was perturbed by bootstrapping rows (with replacement) 10 times.

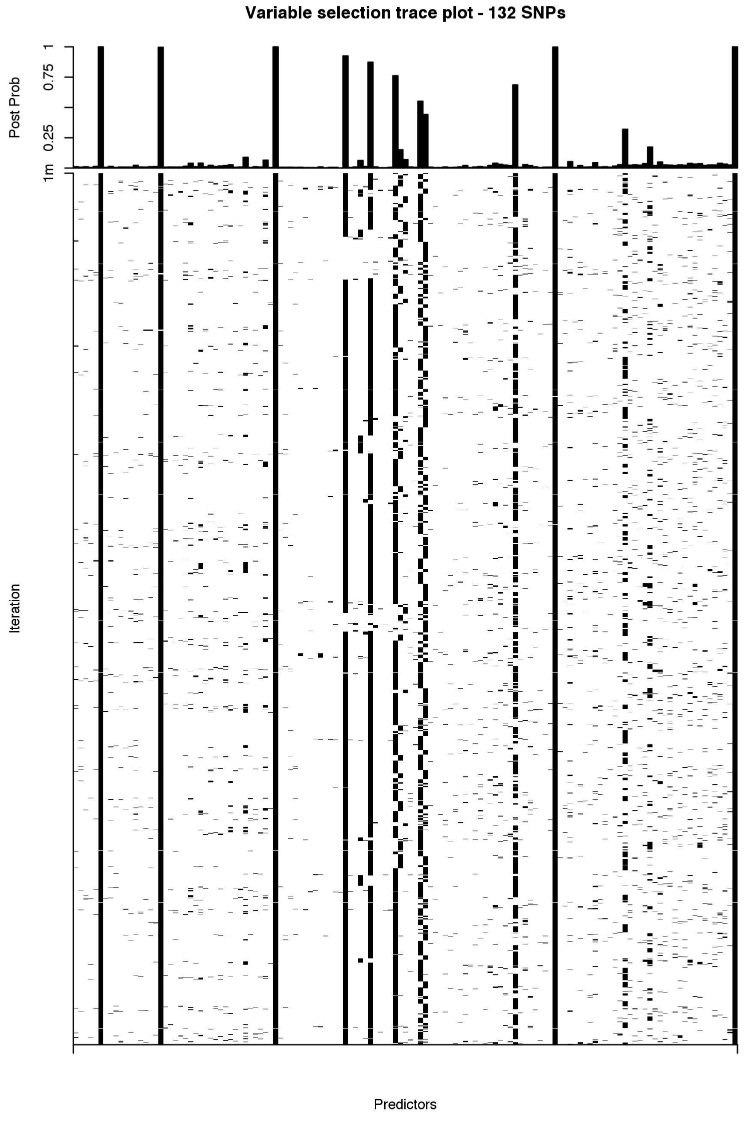

Figure S7: Example trace plot of selection indicators provided by JAM in an analysis of simulated summary statistics from 15,356 individuals (the total size of the MAGIC consortium) for 132 SNPs over 4 regions. Multi-SNP signals (3 effects) were simulated in each region. Predictors are ordered horizontally, and posterior samples from JAM are ordered vertically from bottom to top. For each SNP, inclusion at the particular iteration is denoted in black, and exclusion is denoted in white. This plot helps to visualise the mixing patterns of the selection indicators and if variables seem to stick, i.e. do not come in and out in a fairly regular manner. JAM was run for 2 million iterations.

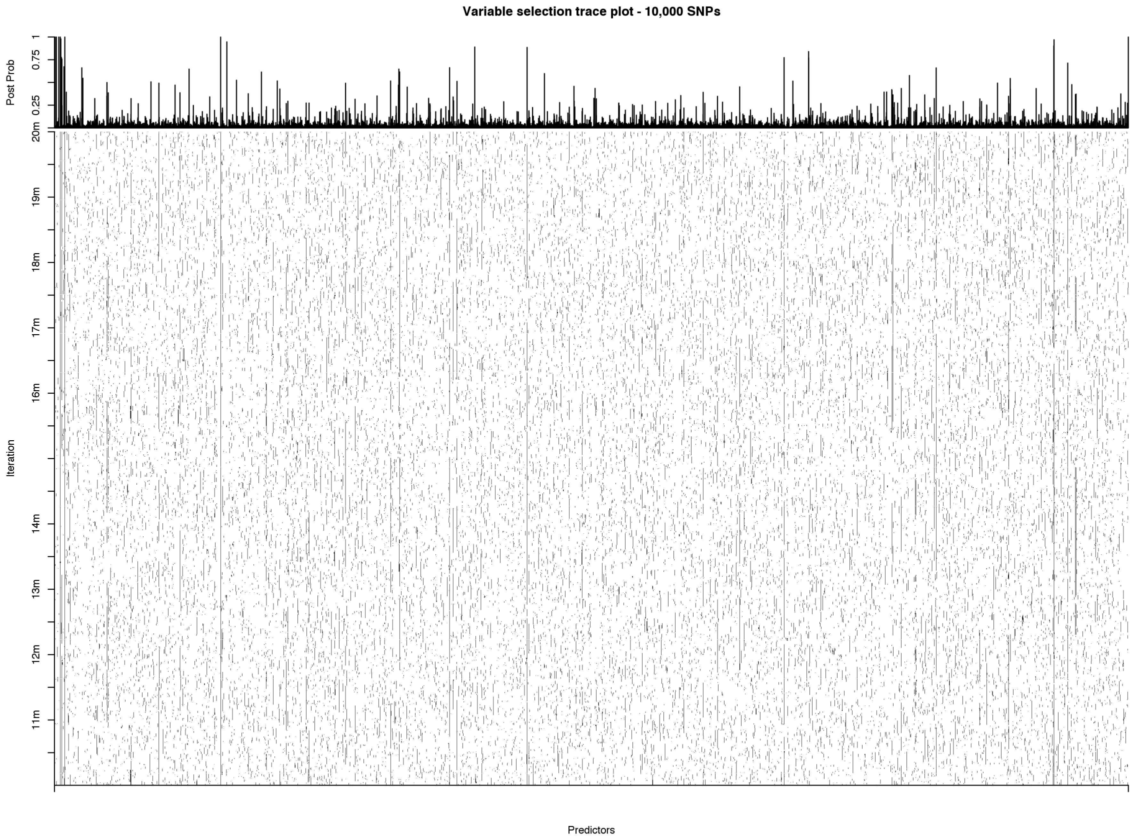

Figure S8: Example trace plot of selection indicators provided by JAM in an analysis of simulated summary statistics from 15,356 individuals (the total size of the MAGIC consortium) for 10,000 SNPs over 304 regions. Multi-SNP signals (3 effects) were simulated in four of the regions. Predictors are ordered horizontally, and posterior samples from JAM are ordered vertically from bottom to top. For each SNP, inclusion at the particular iteration is denoted in black, and exclusion is denoted in white. This plot helps to visualise the mixing patterns of the selection indicators and if variables seem to stick, i.e. do not come in and out in a fairly regular manner. JAM was run for 20 million iterations.

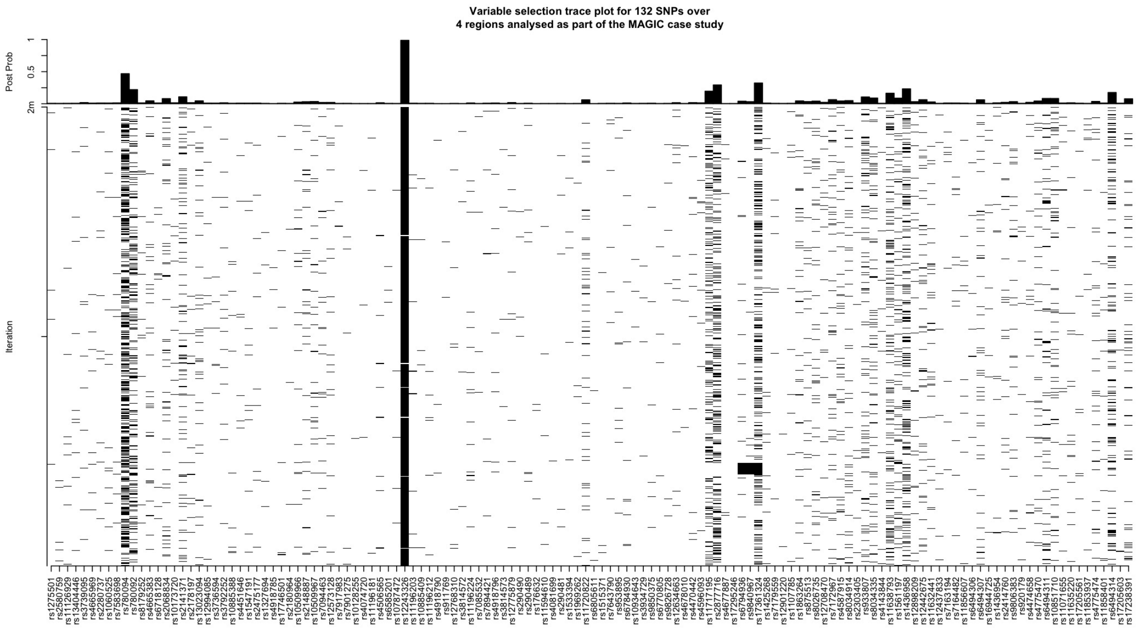

Figure S9: Trace plot of selection indicators provided by JAM in an analysis of summary statistics published by the MAGIC consortium for 132 SNPs over four regions analysed in 15,356 individuals for association with glucose 2 hours after oral stimulation. SNPs are ordered horizontally, and posterior samples from JAM are ordered vertically from bottom to top. For each SNP, inclusion at the particular iteration is denoted in black, and exclusion is denoted in white. This plot helps to visualise the mixing patterns of the selection indicators and if variables seem to stick, i.e. do not come in and out in a fairly regular manner. JAM was run for 2 million iterations. Sticking can be seen during the first million iterations, but note that these iterations (the first half) were discarded as part of the burn-in

References

[1] Green P J. Reversible Jump Markov Chain Monte Carlo Computation and Bayesian Model Determination. *Biometrika*, 82(4):711, 1995.

[2] Yang J, Ferreira T, Morris A P, Medland S E, Madden P a F, et al. Conditional and joint multiple-SNP analysis of GWAS summary statistics identifies additional variants influencing complex traits. *Nature Genetics*, 44(4):369–375, 2012.
